# Transcriptomic and epigenomic analyses revealed that polycomb repressive complex 2 regulates not only developmental but also stress responsive metabolism in *Brassica rapa*

**DOI:** 10.3389/fpls.2023.1079218

**Published:** 2023-02-20

**Authors:** Adji Baskoro Dwi Nugroho, Sujeong Kim, Sang Woo Lee, Dong-Hwan Kim

**Affiliations:** Department of Plant Science and Technology, Chung-Ang University, Anseong, Republic of Korea

**Keywords:** polycomb, H3K27me3, curly leaf, stress response, glucosinolates, *Brassica rapa*

## Abstract

Polycomb group proteins (PcG) play a crucial role in developmental programs in eukaryotic organisms, including plants. PcG-mediated gene repression is achieved by epigenetic histone modification on target chromatins. Loss of PcG components leads to severe developmental defects. CURLY LEAF (CLF), a PcG component in *Arabidopsis*, catalyzes the trimethylation of histone H3 on lysine 27 (H3K27me3), a repressive histone mark in numerous genes in *Arabidopsis*. In this study, we isolated a single homolog of *Arabidopsis* CLF, namely, BrCLF, in *Brassica rapa* ssp. *trilocularis*. Transcriptomic analysis revealed that BrCLF participated in *B. rapa* developmental processes, such as seed dormancy, leaf and flower organ development, and floral transition. BrCLF was also involved in stress signaling and stress-responsive metabolism, such as aliphatic and indolic glucosinolate metabolism in *B. rapa*. Epigenome analysis showed that H3K27me3 was substantially enriched in genes related to these developmental and stress-responsive processes. Thus, this study provided a basis for elucidating the molecular mechanism of the PcG-mediated regulation of development and stress responses in *B. rapa*.

## Introduction

In eukaryotes, a zygote, a single diploid cell, is produced through the successful fertilization between egg and sperm cells. The zygote then undergoes successive cell divisions and differentiation and develops into multi-cellular tissues and organs with distinct functions and characteristics. Even though each cell has the same genomic contents, they differentiate into functionally specialized cells, indicating that well-systematized cell differentiation systems control differential transcriptional control in each cell lineage. However, studies have yet to determine how a single cell can delicately develop into distinctly specialized organs and tissues in multi-cellular organisms such as eukaryotes. Earlier genetic studies using *Drosophila* provided important information and identified polycomb group (PcG) genes, which are genes required for the proper development of the *Drosophila* body plan (Lewis, 1978).

PcG proteins are highly conserved in eukaryotes, including *Drosophila*, mammals, and plants, and they form large multimeric protein complexes, namely, polycomb repressive complexes (PRCs). PRCs are implicated in developmental programs and traditionally divided into PRC2 and PRC1 in eukaryotes (Margueron and Reinberg, 2011). In *Drosophila*, PRC2 has four core components, namely, Enhancer of Zeste (E(z)), Suppressor 12 of zeste (Su(z)12), Extra sex combs (ESC), and Nucleosome remodeling factor 55 (Nurf55) in *Drosophila* (Calonje, 2014). Each PRC2 component has a unique role; specifically, E(z) protein containing a SET domain catalyzes the deposition of a methyl group in histone H3 on lysine 27 (H3K27me3); ESC protein binds to H3K27me2/3 histone marks, thus stabilizing and enhancing the catalytic activity of E(z); Su(z)12 and Nurf55 are required for the nucleosome association of PRC2 to its target chromatin (Cao et al., 2002).

PRC2 components are well conserved in higher eukaryotes, including plants (Kim and Sung, 2014; Huang et al., 2019). *Arabidopsis* genome has three E(z) homologs: CURLY LEAF (CLF: At2g23380), SWINGER (SWN: At4g02020), and MEDEA (MEA, At1g02580); three homologs of Su(z)12, VERNALIZATION 2 (VRN2), EMBRYONIC FLOWRING 2 (EMF2), FERTILIZATION INDEPENDENT SEED 2 (FIS2); a homolog of ESC, FERTILIZATION-INDEPENDENT ENDOSPERM 1 (FIE1); and five homologs of Nurf55, MULTI-SUBUNIT SUPPRESSOR OF IRA 1–5 (MSI1–5) (Chanvivattana et al., 2004).

Three *Arabidopsis* homologs of E(z) (CLF, SWN, and MEA) encode histone methyltransferases involved in PRC2-mediated gene suppression by catalyzing H3K27me3 in target chromatins (Shu et al., 2020). While MEA is required for endosperm development in seeds, SWN plays a partially redundant role with CLF and MEA in development throughout the *Arabidopsis* lifespan (Godwin and Farrona, 2022). Nevertheless, CLF may play a major role in developmental programs in *Arabidopsis* because the null mutant of *CLF* displays severe defects in development, whereas the mutant of *SWN* does not show visible developmental defects compared with that of wild-type plants (Shu et al., 2020). The single CLF mutant, namely, *clf*, displays developmental defects in embryogenesis, seed dormancy, leaf development, floral transition, and floral organ development (Kim and Sung, 2014; Xiao and Wagner, 2015; Yan et al., 2020). For example, the expression of floral integrator genes, such as *FLOWERING LOCUS T* (*FT*) and *SUPPRESSOR OF OVEREXPRESSION OF CO 1* (*SOC1*), was upregulated in the *clf* mutant, which is caused by the loss of the repressive histone mark H3K27me3 in *FT* and *SOC1* chromatin (Jiang et al., 2008; Hou et al., 2014). In addition, the repression of seed dormancy genes, such as *SOMNUS* (*SOM*) and *DELAY OF GERMINATION 1* (*DOG1*), is impaired in the *clf* mutant (Bouyer et al., 2011). Furthermore, the de-repression of homeotic genes, such as *AGAMOUS* (*AG*), *AGAMOUS-LIKE 19 (AGL19*), and *SEPALATTA 3* (*SEP3*), in the *clf* mutant results in abnormal floral organ development (Shu et al., 2019). Therefore, loss of CLF, a catalytic subunit of PRC2, elicits detrimental effects on plant developments, thus showing an upward curly leaf, dwarf phenotype, premature floral transition, embryo lethality, reduced fertility, and deformed floral organs (Goodrich et al., 1997).

Several allelic mutants of *B. rapa* homologs of *Arabidopsis* CLF have been described for Chinese cabbage (*Brassica rapa* L. ssp. *pekinensis*, named as *ebm1* and *ebm3*) and yellow sarson inbred line R-o-18 (*B. rapa* ssp. *trilocularis*, named *braA.clf-1*; Payá-Milans et al., 2019; Huang et al., 2020; Tan et al., 2021). *braA.clf* plants exhibited pleiotropic developmental changes which were resulted from the de-repression of developmental genes *via* the reduction of enrichment of H3K27me3 (Payá-Milans et al., 2019). For example, *braA.clf* plants have a smaller overall size, abnormal floral organs, and upward curling of leaves, resembling to the case of the *Arabidopsis clf* mutant (Goodrich et al., 1997). In addition, enrichment of H3K27me3 histone mark was substantially compromised in the *braA.clf-1* mutant compared with that in wild-type plants (Payá-Milans et al., 2019). All of them are TILLING mutants developed *via* ethyl methanesulfonate (EMS) mutagenesis treatment. They commonly exhibit similar phenotypic defects such as upward curly leaves, reduced plant height, altered floral organs, and premature flowering phenotype observed in *Arabidopsis*. The enrichment of H3K27me3 histone mark is substantially compromised in the *braA.clf-1* mutant compared with that in wild-type plants. Another EMS screening also isolated a mutant named as’tu8’ displayed a small malformation of shoot organs and reduced level of indole glucosinolates (GSLs). The *TU8* was identified as an allele of *TERMINAL FLOWER 2* (*TFL2*) (also referred to as *LIKE-HETEROCHROMATIN 1* (*LHP1*)) that encodes a homeodomain protein functioning with PRC1 and PRC2 complexes (Ludwig-Müller et al., 1999).

Even though *B. rapa* CLF is required for several facets of developmental programs of *B. rapa*, the functional importance of BrCLF is poorly understood. In this study, a null mutant named *brclf* (same allele previously known as *braA.clf-1*) was isolated as a *B. rapa* CLF homolog of *Arabidopsis* CLF. Genome-wide transcriptomic, epigenomic, and metabolic analyses were performed to understand the functional significance of *BrCLF* in *B. rapa*. Thus, this study revealed that BrCLF-containing PRC epigenetically regulated developmental and stress response programs in *B. rapa*.

## Materials and methods

### Plant materials and growth conditions

Seeds of *B. rapa* yellow sarson (ssp. *trilocularis*) inbred line R-o-18 (Rusholme et al., 2007) and *brclf* mutant were sterilized with 5% sodium hypochlorite solution and then washed with distilled water. Sterilized seeds were plated on 1/2 Murashige and Skoog (MS) media and stored in a refrigerator at 4°C for 3 days for stratification. Two-week-old seedlings were transplanted to plastic pots containing vermiculite soil and watered with tap water. The plants were grown in a growth room under a long-day condition (16 h/8 h cycle) at 22°C with cool-white fluorescent illumination (120 mol m^-2^ s^-1^, FHF32SSEX-D fluorescent tube; Osram, South Korea). Phenotypic analysis was performed to compare ‘R-o-18’ and ‘*brclf’* during vegetative and reproductive stages. A total of 8 plants were used from each ‘R-o-18’ and ‘*brclf*’ to measure hypocotyl, leaf, and rosette at 4-weeks after germination. Meanwhile, 5 plants were used in the measurement of plant height, petal, bud, and pistil at 2-weeks after bolting. The number of siliques and seeds was also quantified in this study at 2-3weeks after ‘R-o-18’ and ‘*brclf*’ flowering.

### Characterization of *brclf* mutant

An EMS-induced TILLING mutant *brclf* (line number: ji32391-a) was purchased from RevGenUK (Stephenson et al., 2010). Genomic DNA from individual *brclf* mutants were extracted using DNeasy Plant mini kit (QIAGEN, Germany) and used for PCR amplification. Gene-specific primer pair (BrCLF_genoF and BrCLF_genoR) were used for genotyping of *brclf* mutant using T1000™ thermal cycler (Bio-rad, USA). Amplified PCR fragments (about 500bp) were run on 1% agarose gel and extracted from gel by using a QIAquick Gel extraction kit (QIAGEN, Germany) and then confirmed by the direct Sanger sequencing (Bionics Co., South Korea). Information on primer sequences is presented in **Supplementary Table S1**.

### Quantitative RT-PCR expression analysis

Total RNAs from the whole seedling of 2-week-old *B. rapa* plants were purified using an RNeasy Plant mini kit in accordance with the manufacturer’s instructions (QIAGEN, Germany). Extracted RNAs were further treated with DNase I (New England Biolabs, USA) for 20 mins at 37°C to eliminate genomic DNA contamination. Purified RNAs were used to synthesize the complementary DNA synthesis using EasyScript RTase (TransGen Biotech, China). Quantitative RT-qPCR (qRT-PCR) reactions were performed using Sol™ 2X Real-Time PCR Smart mix under the following cycling conditions: 95°C for 10 mins followed by 45 cycles of 95°C for 20s, 60°C for 25 s, and 72°C for 35 s. *BrPP2Aa* (Bra012474) was used as the reference gene (Kim et al., 2022). qRT-PCR reactions were performed with three technical replicates by using the LineGene 9600 Plus (FQD-96A) Real-Time PCR Detection System (BioER, China). The detected quantification cycle (cq) values were examined using the 2^–ΔCT^ method to calculate changes in gene expression. To ensure the reliability of quantitative analysis with standard deviation error bars. The primer sequences were designed on the basis of sequence information from the *Brassica* database (BRAD) (Chen et al., 2022) and summarized in the **Supplementary Table S1**.

### Multiple sequence alignment and phylogenic analysis

The nucleotide and amino acid sequences of *BrCLF* were compared using the multiple sequence alignment webtool MultAlin (http://multalin.toulouse.inra.fr/multalin/) (Corpet, 1988). For phylogenic analysis, the amino acid sequences of AtCLF and AtCLF-like gene (SWN and MEA) in *Arabidopsis* genome were obtained from the TAIR database (http://www.arabidopsis.org). The sequence information of *B. rapa* homologs of CLF-like proteins were searched and downloaded from *B. rapa* genome database (http://brassicadb.cn/#/). The protein sequence information of *Arabidopsis* and *B. rapa* homologs were combined in fasta format and submitted into ClustalX (ver. 1.81) sequence alignment program.

### RNA-seq library sequencing

Three biological replicates for 2-week-old seedlings of R-o-18 and *brclf* mutant were harvested and frozen in liquid nitrogen. Total RNAs were extracted using an RNeasy Plant mini kit (QIAGEN, Germany). The quantity and quality of RNA were checked with a 2100 Bioanalyzer (Agilent, USA), and only the samples with an RNA integrity number of >7 were used for library preparation. A library was constructed using TruSeq Stranded mRNA LT Sample Prep Kit (Illumina Inc., USA) in accordance with the manufacturer’s instructions. The constructed RNA-seq libraries were sequenced on a NovaSeq 6000 platform system (Macrogen Co., South Korea). Paired-end sequencing protocol was employed.

### RNA-seq alignment and analysis

The quality of RNA-seq read counts was first evaluated using the FastQC software (http://www.bioinformatics.babraham.ac.uk/projects/fastqc). On the basis of FastQC results, raw reads were trimmed and quality-filtered before genome alignment by using Trimmomatic (ver0.36; Bolger et al., 2014). The trimmed and filtered reads with more than 90% portion (Q>30) were only used for mapping. The reference genome of *B. rapa* plants was obtained from Ensembl (https://plants.ensembl.org/info/website/ftp/index.html). Mapping was performed using the TopHat2 software with default parameters (Kim et al., 2013). Aligned reads were converted to digital counts using FeatureCounts (Liao et al., 2014) and were analyzed using edgeR (Robinson et al., 2010). Differentially expressed genes (DEGs) were identified on the basis of p < 0.05 and two-fold difference cut-off. A multi-dimensional scaling (MDS) plot and correlation heap were generated using R packages (ver. 3.6.0) (R Core Team, 2020) (https://www.R-project.org/). Venn diagram analysis was performed using VENNY (v2.1) webtool (https://bioinfogp.cnb.csic.es/tools/venny/). GO analysis was conducted using ShinyGO (ver 0.76) program (Ge et al., 2020). Aligned reads were visualized with the Integrative Genomics Viewer (IGV) program of Broad Institute (Thorvaldsdóttir et al., 2013). Heatmap analysis was performed using multi experiment viewer (MEV) program (ver 4.9.0).

### Determination of glucosinolate content through U-HPLC analysis

Glucosinolates were analyzed from 2-week-old R-o-18 and *brclf* mutant in accordance with a previously described protocol (Nugroho et al., 2021). They were extracted from whole fresh tissue samples with 70% aqueous methanol (methanol:water 70:30, v:v) at 70°C for 10 min. The extract was centrifuged at 3000 × *g* for 20 min, and the supernatant was transferred into a column containing Sephadex A-25 (Sigma-Aldrich Inc., USA). The column was reacted with 11.25 units of purified sulfatase (Sigma-Aldrich Inc., USA) at 37°C to allow desulfation for 12 h. Desulfo-glucosinolates (DS-GSLs) were eluted from the column with 1.5 ml of deionized water and evaporated using a speed vacuum. DS-GSLs were re-dissolved with 1 ml of HPLC water and filtered with 0.45 μm PVDF membrane (Biofact, Korea). They were chromatographically separated on a C18 reverse phase column (Zorbax XDB-C18, 4.6 × 250 mm^2^, 5 μm particle size, Agilent, USA) with a gradient system composed of water (Thermo Fisher Scientific, USA) and acetonitrile (Honeywell, USA) in the Dionex Ultimate 3000 ultra-high performance liquid chromatography (U-HPLC) systems (Thermo Fisher Scientific, USA). Samples (20 μL) of DS-GSLs were analyzed using a diode array detector at 229 nm. All DS-GSL peaks detected in this study were identified in accordance with a previous study (Nugroho et al., 2019). Each DS-GSL was independently quantified from three biological replicates based on sinigrin (Sigma-Aldrich Inc., USA) standard compounds (Brown et al., 2003). Data were presented as micromoles per gram dry weight (µmol/gr DW).

### Analysis of H3K27me3 ChIP-seq dataset

The dataset of the genome-wide H3K27me3 profile (NCBI SRA number: PRJNA542357) in leaf and inflorescence tissues of R-o-18 inbred line (*B. rapa* ssp *trilocularis*) was downloaded. The reference genome of *B. rapa* was also downloaded from Ensembl Plants database (https://plants.ensembl.org/). The downloaded raw fastq files of H3K27me3 were initially quality-checked using the FastQC program (Andrews, 2010). On the basis of FastQC result, low-quality reads were trimmed and filtered using Trimmomatic (v0.36) (Bolger et al., 2014), and the filtered fastq files were used for mapping to *B. rapa* genome by using bowtie2 (Langmead and Salzberg, 2012). SAMtools (Li et al., 2009) were used to convert SAM files to BAM files. Then, duplicated reads were removed by Picard MarkDuplicates (v2.18.2.0; biotools:picard_tools; RRID : SCR_006525). Deduplicated reads were then used for peak calling on pooled replicates by using MACS2 (v2.1.1) for comparison between input DNA and ChIP samples. The distribution of H3K27me3 enrichment was visualized using the IGV (Thorvaldsdóttir et al., 2013).

### Analysis of ChIP-qPCR

The 2-weeks seedlings of ‘R-o-18’ and ‘*brclf*’ were cross-linked with 1% formaldehyde solution under vacuum for 25 min and stop the reaction by addition of 0.1M glycine. The cross-linked seedlings were dried and grinded in liquid nitrogen. ChIP experiment was performed as previously reported. Ten micrograms of monoclonal antibody against H3K27me3 histone mark (ab6002, Abcam, United Kingdom) were treated for the individual ChIP sample. The immunoprecipitated and input DNAs were used for the qPCR analysis. ChIP-qPCR reactions were performed using Sol™ 2X Real-Time PCR Smart mix (SolGent, Korea) under the following cycling conditions: 95°C for 12 mins followed by 45 cycles of 95°C for 20s, 60°C for 25 s, and 72°C for 35 s. *BrFUS3* (Bra032953) was used as the reference gene.

### Statistical analysis

Data were statistically analyzed using a statistical software package (SAS; version 9.4; SAS Institute Inc., Cary, NC, USA). Statistical differences were calculated through one-way analysis of variance (ANOVA) and *post-hoc* Tukey’s test (p < 0.05). Data were presented as means ± standard deviation (SD) of three biological replicates.

## Results

### Phenotypic characterization of *brclf* mutant

In *Arabidopsis*, CURLY LEAF (CLF) plays a catalytic activity in PRC2 for gene suppression *via* the trimethylation of histone H3 at the K27 residue (H3K27me3) (Schubert et al., 2006). *B. rapa* genome has a single homolog of *Arabidopsis CLF* referred to as *BrCLF* (Bra032169) from the *Brassica* database (BRAD) (Payá-Milans et al., 2019; **Supplementary Figure S1A**). Because *Arabidopsis* CLF has two paralogs, namely, *SWINGER* (*SWN*) and *MEDEA* (*MEA*), in the genome, we searched homologs of these paralogs from the *B. rapa* genome database through BLAST search and phylogenic analysis. We found that *B. rapa* has one homolog of *SWN* (named *BrSWN*, Bra036300) and two homologs of *MEA* (*BrMEA*,*a* [Bra033334] and *BrMEA*,*b* [Bra032592]; **Supplementary Figure S1B**).

**Figure 1:**
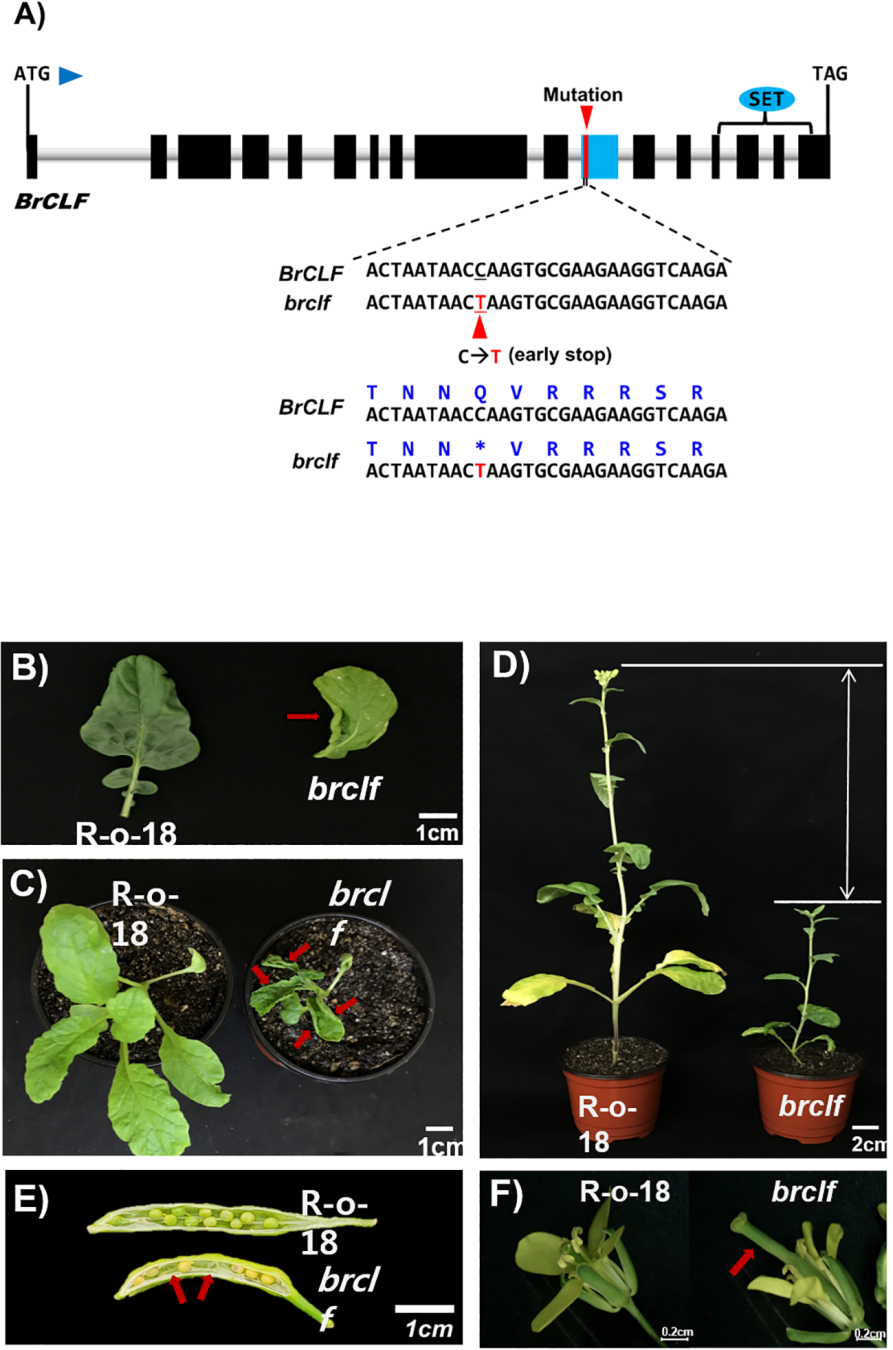
Phenotypic characterization between R-o-18 wild type and *brclf* mutant. **(A)** Identification of point mutation in the *brclf* coding sequence that generates a premature stop codon. The conversion of cytosine to thymine at 1,843 bp from the start codon was indicated with an upward red arrowhead. Glutamine (Q) amino acid at the 615th residue of BrCLF of R-o-18 wild type was converted to a stop codon (indicated with an asterisk) in the *brclf* mutant. **(B)** Comparison of leaf phenotypes between R-o-18 and *brclf* mutant. The *brclf* mutant exhibited a smaller and upward curly leaf phenotype than R-o-18 did. The red arrow indicates the upward curly leaf of the *brclf* mutant. **(C)** Whole plant phenotype of four-week-old R-o-18 and *brclf* mutant. The *brclf* mutant showed smaller rosette diameter and curly leaves (indicated with red color arrows) than the wild-type R-o-18 did. **(D)** Phenotypic comparison of R-o-18 and *brclf* at the flowering stage. Flowering occurred earlier in the *brclf* mutant than in R-o-18; furthermore, the height of the former was shorter than that of the latter. **(E)** Comparison of seed sets between R-o-18 and *brclf* mutant. *brclf* showed lower number of seeds and some aborted seeds (indicated with red color arrows) in a pod compared with R-o-18. **(F)** Comparison of flower organ between R-o-18 and *brclf*. *brclf* showed an abnormally elongated pistil compared with R-o-18 (indicated with red color arrow).

CLF plays a crucial role in developmental programs, including leaf development and floral organ development, in *Arabidopsis* (Goodrich et al., 1997). Thus, we aimed to obtain the loss-of-function mutant of *BrCLF* by searching an EMS-mutagenized TILLING mutant population in *B. rapa* ssp. *trilocularis* (hereafter referred to as *brclf* mutant). Point mutation (C to T) in the 1,843 bp region from the start codon of *BrCLF* was detected to produce a premature stop codon (Gln at the 615th residue from the start codon) in *brclf* mutant (**Figure 1A**; **Supplementary Figure S1C**). This mutation led to the loss of 292 amino acids in the C-terminal region, which has a catalytic SET domain (**Supplementary Figure S1D**). Non-functional *brclf* mutant exhibited severe developmental defects in vegetative and reproductive organs compared with those in the wild type (WT) R-o-18 plants. For example, the *brclf* mutant showed abnormal phenotypes such as upward curled and smaller leaves than the R-o-18 wild-type plant did (**Figures 1B, C**). The hypocotyl length, leaf width, rosette diameter of *brclf* mutant were significantly shorter than those of R-o-18 in 4-week-old vegetative-stage plant in our growth condition (**Supplementary Figure S2A**). At the reproductive stage, *brclf* was quite shorter than the wild-type plant (**Figure 1D**; **Supplementary Figure S2B**). Seed fertility was significantly reduced in *brclf* mutant than the wild-type plant (**Figure 1E**; **Supplementary Figure S2B**). A lower seed set in *brclf* mutant might cause by the deformed development of floral organs (**Figure 1F**). For instance, the pistil of *brclf* was extraordinarily elongated, which might substantially reduce the chance for pollination and successive fertilization between egg and sperm cells.

### Transcriptomic change in *brclf* mutant

**Figure 2:**
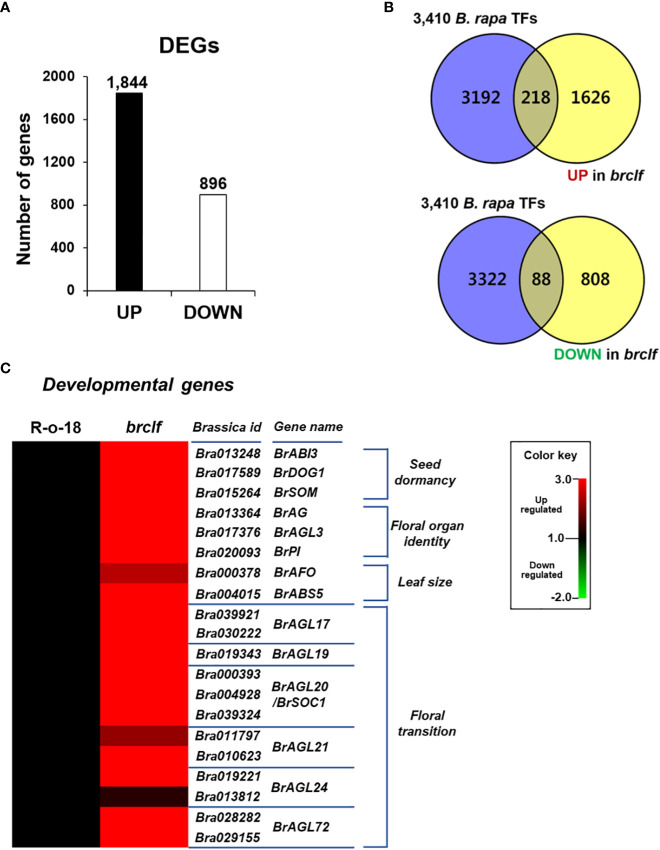
Identification of differentially expressed genes (DEGs) between “R-o-18-and *brclf*. **(A)** Number of DEGs between “R-o-18-and *brclf*. A total of 1,844 and 896 genes were respectively upregulated and downregulated in the *brclf* mutant compared with those in wild-type R-o-18. **(B)** Venn diagram showing the overlap between 3,540 TF genes and DEGs found in *brclf* in comparison with R-o-18 in *B. rapa*. A total of 218 and 88 TFs were upregulated and downregulated in *brclf* compared with those in R-o-18, respectively. **(C)** Heatmap showing normalized transcript levels of 20 TF genes related to developmental programs in *B. rapa*: 3 genes (*BrABI3*, *BrSOM*, *BrDOG1)* related to “seed dormancy,” 3 genes related to the “floral organ identity” (*BrAG*, *BrAGL3*, *BrPI*), 2 genes related to “leaf size” (*BrAFO*, *BrABS5*), and 12 genes related to the “floral transition” (*BrAGL17.1/2*, *BrAGL19*, *BrAGL20.1/2/3*, *BrAGL21.1/2*, *BrAGL24.1/2*, and *BrAGL72.1/2*).

The *brclf* mutant exhibited severe developmental defects. Thus, we aimed to dissect the transcriptomic change between WT and *brclf* mutant by RNA-seq analysis. After low-quality reads were trimmed, high-quality paired reads (>Q30) were over 95% in all samples (**Supplementary Table S2**). They were used for alignment with the *B. rapa* reference genome downloaded from the Ensembl Plants (https://plants.ensembl.org/info/data/ftp/index.html). Correlation heatmap analyses showed a distinct clustering between R-o-18 and *brclf* samples (**Supplementary Figure S3A**), indicating that RNA-seq libraries were well constructed and sequenced. Differentially expressed genes (DEGs) between R-o-18 and *brclf* samples were isolated based on the pairwise sample comparisons using edgeR (**Figure 2A**; **Supplementary Figure S3B**). A total of 2,740 DEGs, including 1,844 upregulated genes and 896 downregulated genes in *brclf* mutant compared with wild type R-o-18, were detected as significant DEGs in pairwise sample comparisons (|log_2_|> 1, p < 0.05) (**Supplementary Table S3**).

### BrCLF is required to regulate genes related to multiple developmental transitions in *B. rapa* L.

Because *Arabidopsis* CLF was reported to regulate thousands of genes related to various developmental processes, we examined how many transcription factors are transcriptionally affected in *brclf* in comparison to R-o-18 sample, we first searched the BRAD genome database and collected total 3,410 transcription factor genes in *B. rapa* genome. Among 3,410 TFs, total 218 and 88 TF genes were up- and downregulated in *brclf* compared with R-o-18, respectively (**Figure 2B**; **Supplementary Table S4**). Given that BrCLF-containing PRC2 exerts a repressive role in target gene transcription, the number of upregulated genes in *brclf* was possibly higher (2.5 times) than that of downregulated genes. The expression of several key TF genes involved in seed dormancy/germination, leaf development, floral organ identity, and floral transition were examined between R-o-18 *and brclf*. Twenty genes related to developmental processes were selected, and their transcript levels were compared between R-o-18 and *brclf*: 3 genes related to “seed dormancy” such as *BrABI3* (*B. rapa* homolog of *ABI3*, Bra013248), *BrDOG1* (*B. rapa* homolog of *DOG1*, Bra017589), *BrSOM* (*B. rapa* homolog of *SOM*, Bra015264), 3 genes associated with “floral organ identity,” *BrAG* (*B. rapa* homolog of *AG*, Bra013364), *BrAGL3* (*B. rapa* homolog of *AGL3*, Bra017376), and *BrPI* (*B. rapa* homolog of *PI*, Bra020093), 2 genes related to “leaf size” (*BrAFO*, *B. rapa* homolog of *AFO*, Bra000378 and *BrABS5*, *B. rapa* homolog of *ABS5*, Bra004015), and 12 *BrAGL* genes related to the “floral transition” (*BrAGL17*, *BrAGL19*, *BrAGL20/SOC1*, *BrAGL21*, *BrAGL24*, and *BrAGL72*). All tested *B. rapa* developmental genes were upregulated in *brclf* in comparison with those of the R-o-18 wild type (**Figure 2C**). This result indicated that BrCLF (and its PRC2 complex) play a negative role in the expression of many key developmental TF genes in *B. rapa* plant. Quantitative RT-PCR (qRT-PCR) was used to determine the transcript levels of these TF genes between R-o-18 and *brclf* mutant and validate the RNA-seq results. In a consistency with RNA-seq result, qRT-PCR analysis showed that transcript levels of all tested developmental genes were substantially upregulated in *brclf* mutant compared with the wild type R-o-18 plant (**Supplementary Figure S4**). Altogether, our RNA-seq and qRT-PCR data confirmed that BrCLF play a repressive role in transcription of key developmental genes in *B. rapa* plant.

### Gene ontology analysis of DEGs between R-o-18 and *brclf*

**Figure 3:**
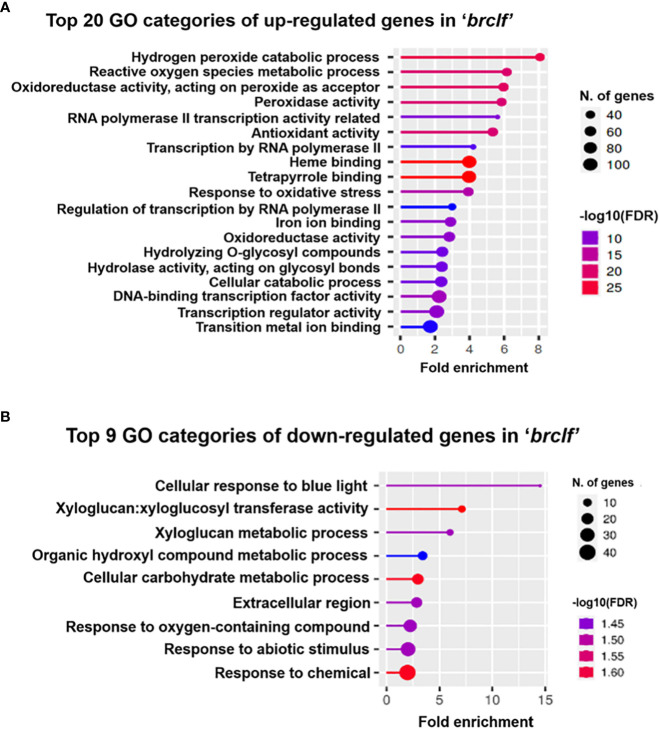
Gene ontology (GO) analysis of upregulated and downregulated genes in *brclf* mutant compared with those in R-o-18 wild-type plant. **(A)** Top 20 functional GO categories with 1,844 upregulated genes in the *brclf* mutant compared with those in the R-o-18 wild-type plant. **(B)** Top 9 functional GO categories with 896 downregulated genes in the *brclf* mutant compared with those in the R-o-18 wild-type plant.

Besides developmental TF genes, thousands of genes were differentially expressed between R-o-18 and *brclf*. GO analysis was conducted using ShinyGO v. 0.76 (http://bioinformatics.sdstate.edu/go/) to further seize the biological role of BrCLF from the list of DEGs. GO analysis using the list of upregulated or downregulated genes displayed top 20 and top 9 GO categories, respectively (**Figure 3**). Unexpectedly, GO analysis using the list of upregulated genes exhibited that the top rankers in the top 20 categories were enriched with stress-related categories such as “hydrogen peroxide catabolic process” and “reactive oxygen species metabolic process” (**Figure 3A**). As expected, GO categories related to “regulation of transcription” were also shown in the top 20 categories, but they were in lower ranks. GO analysis was also conducted with the lists of downregulated genes (**Figure 3B**). GO analysis using list of downregulated genes showed the enrichment of GO categories related to metabolic processes such as “cellular response to blue light,” “xyloglucan metabolism,” “carbohydrate metabolism,” “response to abiotic stimulus,” and “response to oxygen-containing compound.” Based on the repressive role of PRC2 complex in gene regulation, it is likely that a majority of GO terms found with downregulated genes might not be the directly targeted functional categories. Results of GO analysis using DEGs suggested that BrCLF might play a regulatory role on the stress signaling genes involved in the “hydrogen peroxide catabolic process” and “reactive oxygen species metabolic process” in *B. rapa*. Based on this observation, we further analyzed the list of DEGs containing 1,844 up-regulated and 896 down-regulated genes. We collected 3,150 stress related genes from the *Arabidopsis* genome, which was obtained from the STIFDB2 (Stress-responsive Transcription Factor Database, ver. 2) website (http://caps.ncbs.res.in/stifdb2/index.html). Based on the sequence homolog search results between *Arabidopsis* and *B. rapa*, we found a total of 5,226 stress-responsive genes in *B. rapa* (**Supplementary Table S5**). Out of 5,225 stress-related genes, 301 genes were found in the 1,844 up-regulated genes in ‘*brclf*’ (**Supplementary Figure S5**; **Table S6**). It indicated that 16% (301/1,844) of stress-related genes were affected in the ‘*brclf*’ mutant.

### BrCLF is involved in the regulation of plant stress signaling genes in *B. rapa* L.

**Figure 4:**
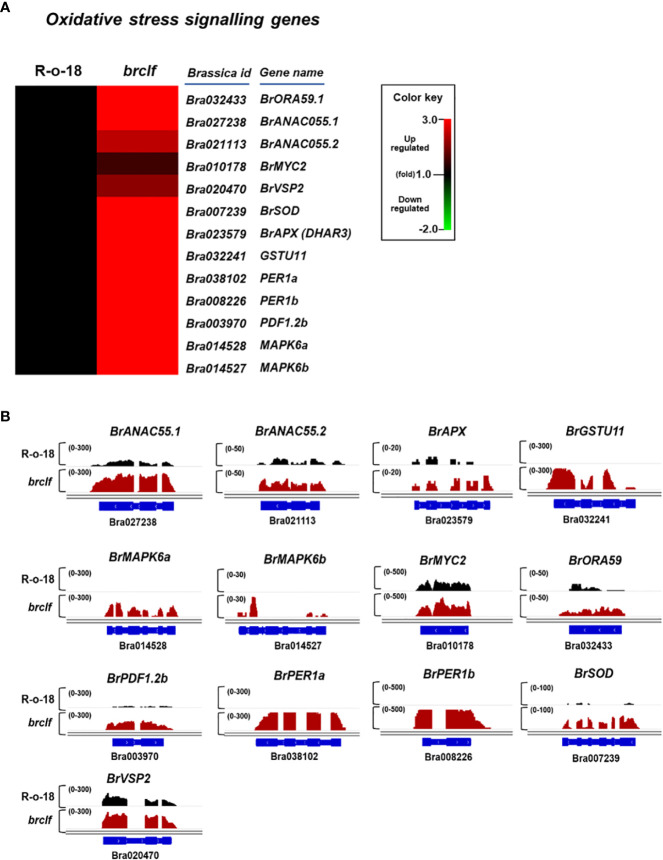
Expression of oxidative stress-related genes between R-o-18 and *brclf*. **(A)** Heatmap showing the normalized transcript levels of 13 oxidative stress-related genes in *B. rapa*. Transcript level of R-o-18 was set to value 1 and relative transcript level of each gene in *brclf* was presented. **(B)** Integrative genome browser (IGV) illustration on transcript levels of 13 stress signaling genes between ‘R-o-18’ and ‘*brclf*’. *BrPP2Aa* was used as the reference gene to confirm the normalization of RNA-seq reads between ‘‘R-o-18’ and ‘*brclf*’. Black colors and red colors indicates transcript reads mapped to individual genes in ‘R-o-18’ and ‘*brclf*’, respectively. Read coverage normalized by total number of mapped reads are indicated at the top left corner of each track in parentheses.

Because GO enrichment suggested a possible role of BrCLF on stress signaling, we selected 13 genes related to “hydrogen peroxide catabolic process” and “reactive oxygen species metabolic process” response from *B. rapa* genome (**Supplementary Table S7**). It included *B. rapa MYC2* (*BrMYC2*, Bra010178), *B. rapa OCTADECANOID-RESPONSIVE ARABIDOPSIS AP2/ERF 59* (*BrORA59*, Bra010178), *B. rapa NAC DOMAIN CONTAINING PROTEIN 55* (*BrANAC55*), *B. rapa VEGETATIVE STORAGE PROTEIN 2* (*BrVSP2*, Bra020470), *B. rapa MANAGANESE SUPEROXIDE DISMUTASE* (*BrSOD*, Bra007239), *B. rapa ASCORBATE PEROXIDASE* (*BrAPX*, Bra023579), *B. rapa GLTATHIONE S-TRANSFERASE 11* (*BrGSTU11*, Bra003970), *B. rapa PEROXIDASE 1b* (*BrPER1b*, Bra032241), *B. rapa PEROXIDASE 1a* (*BrPER1a*, Bra038102), *B. rapa PLANT DEFENSIN 1.2b* (*BrPDF1.2b*, Bra003970), *B. rapa MAP KINASE 6a* (*BrMPK6a*, Bra014528), *B. rapa MAP KINASE 6b* (*BrMPK6b*, Bra014527) homologs. The analysis of RNA-seq data showed that transcript levels of these 13 genes were commonly higher in *brclf* sample than those of R-o-18 sample (**Figure 4A**). The qRT-PCR analysis of these genes also confirmed the result of RNA-seq (**Supplementary Figure S6**). These results suggested that BrCLF is involved in the suppression of stress signaling genes in *B. rapa*.

### BrCLF regulates genes related to stress-responsive “glucosinolate metabolism”

In plants, “oxidative stress,” “hydrogen peroxide metabolism” and “reactive oxygen species (ROS) responses are initial signaling events upon exposure to a diversity of stresses such as biotic (i.e., insect and pathogen attack) and abiotic stress (i.e., salinity, heat and drought) (Nazir et al., 2020). Stress-induced signaling cascade stimulates the accumulation of defensive secondary metabolites such as aliphatic and indolic glucosinolates (abiotic and biotic stress metabolites) (Kliebenstein, 2004; Hiruma et al., 2013; Meraj et al., 2020). Thus, we decided to examine whether BrCLF is involved in the regulation of genes related to these stress-responsive secondary metabolisms, glucosinolates. Glucosinolates (GSLs) are a group of sulfur-rich compounds present in Brassicaceae family crop plants including *Brassica* genus plants (i.e., *B. rapa*.) (Sonderby et al., 2010). We found total 104 GSL pathway genes (63 aliphatic and 41 indolic GSL genes) from the *B. rapa* genome (**Supplementary Table S8**). Among the list of 1,844 up-regulated genes, total 9 GSL pathway genes were significantly upregulated (fold change ≥2) in comparison to levels of ‘R-o-18’ (**Supplementary Table S9**). In addition, additional 18 GSL pathway genes were moderately up-regulated genes (fold change ≥1.5 & <2) in ‘*brclf*’ mutant compared with R-o-18. Thus, total 27 out of 104 genes (26%) were upregulated in *brclf*, whereas none GSL pathway genes was downregulated in ‘*brclf*’ in comparison to levels of R-o-18 (fold change cutoff ≥1.5).

**Figure 5:**
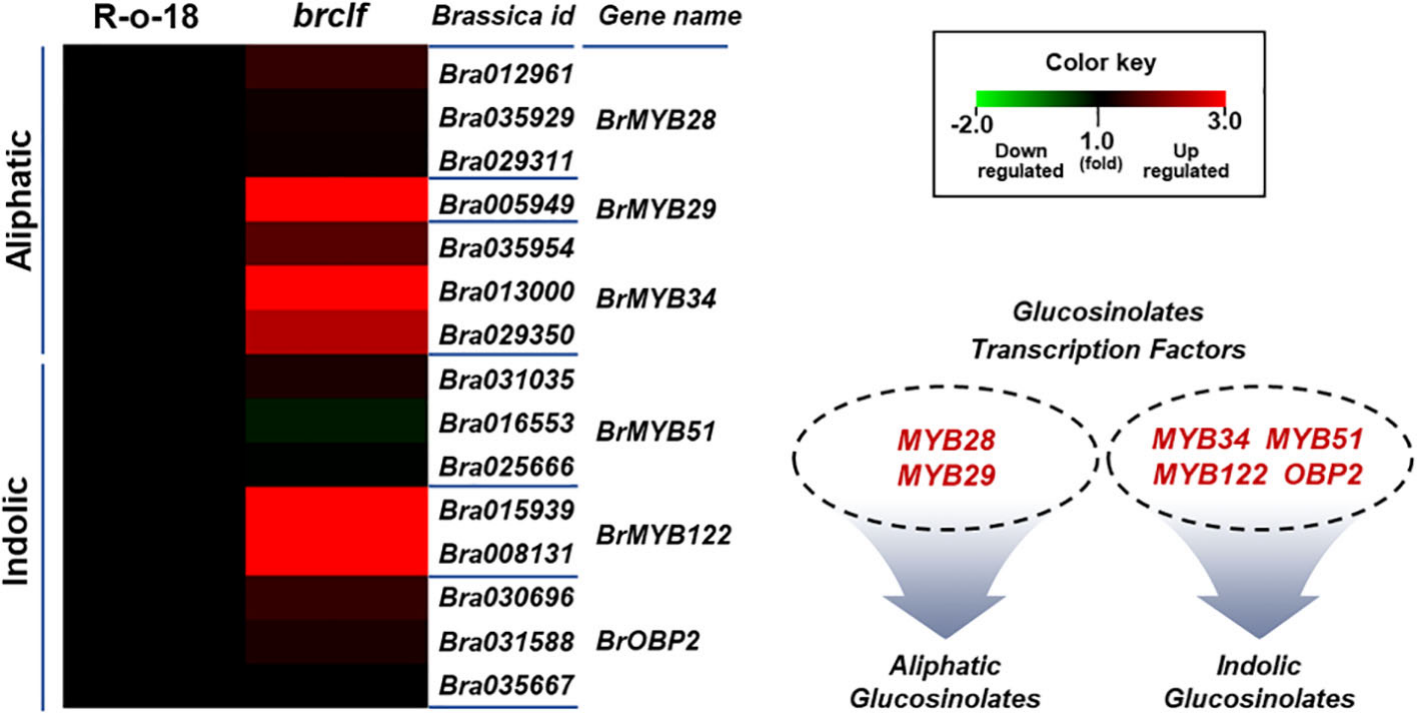
Expression levels of 15 TFs controlling aliphatic and indolic GSL biosynthesis genes between R-o-18 and *brclf*. Heatmap showing expression levels of 4 TFs (*BrMYB28.1–3 and BrMY29*) controlling aliphatic GSL biosynthesis genes and 11 TFs (*BrMYB34.1–3*, *BrMYB51.1–3*, *BrMYB122.1–2*, and *BrOBP2.1–3*) *controlling indolic GSL biosynthesis gene* factors between R-o-18 and *brclf*. Transcript level of R-o-18 was set to value 1, and relative transcript level of *brclf* was presented.

**Figure 6:**
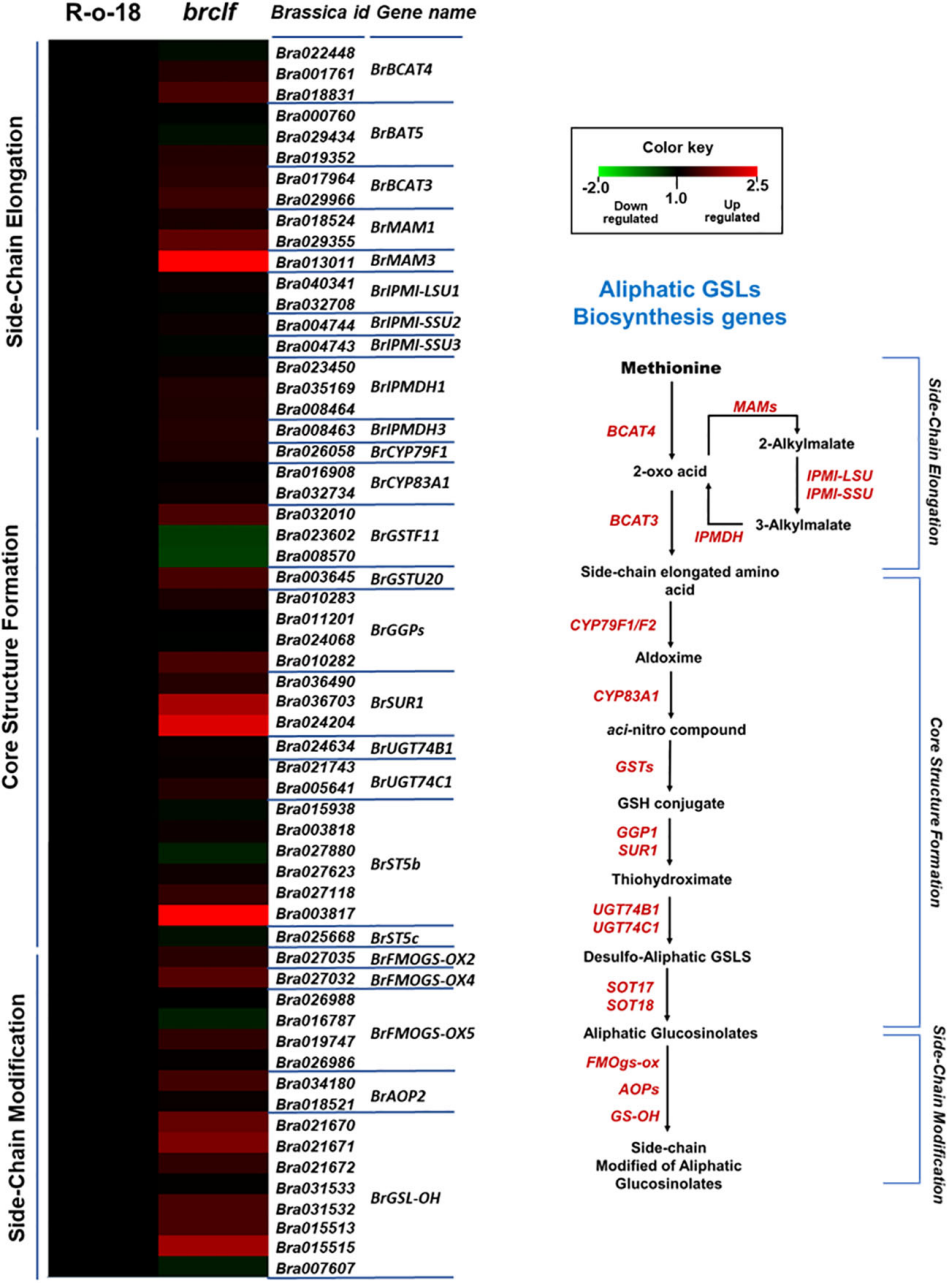
Expression levels of 59 aliphatic GSL biosynthesis genes between R-o-18 and *brclf*. **(A)** Heatmap showing expression levels of 59 aliphatic GSL biosynthesis genes between R-o-18 and *brclf*. Transcript level of R-o-18 was set to value 1, and relative transcript level of *brclf* was presented. **(B)** Diagram showing biosynthetic pathway for aliphatic GSL compounds. Genes responsible for each catalytic conversion were indicated with red letters.

**Figure 7:**
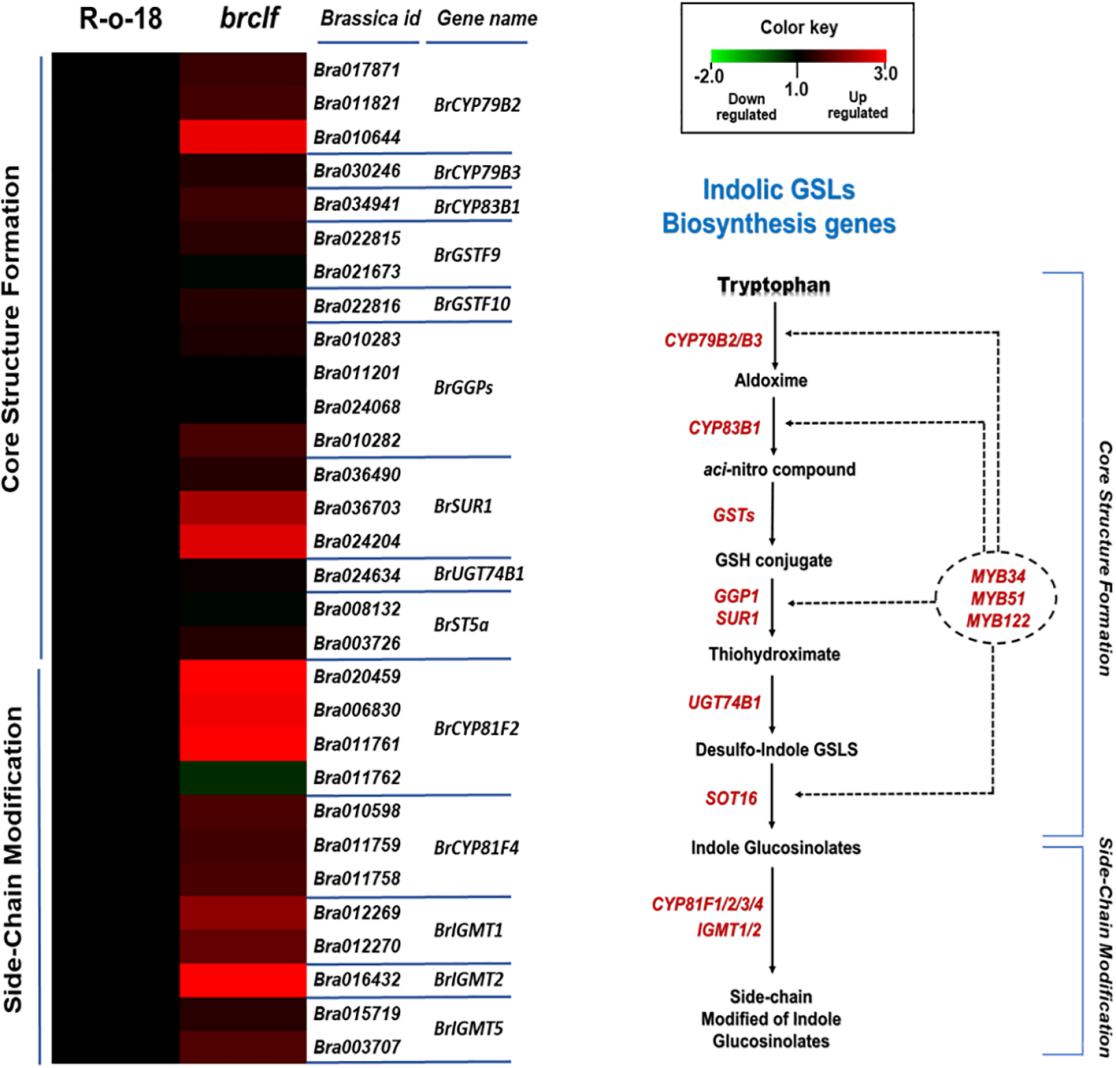
Expression levels of 40 indolic GSL biosynthesis genes between R-o-18 and *brclf*. **(A)** Heatmap showing the expression levels of 40 indolic GSL biosynthesis genes between R-o-18 and *brclf*. Transcript level of R-o-18 was set to value 1, and relative transcript level of *brclf* was presented. **(B)** Diagram showing biosynthetic pathway for indolic GSL compounds. Genes responsible for each catalytic conversion were indicated with red letters.

**Figure 8:**
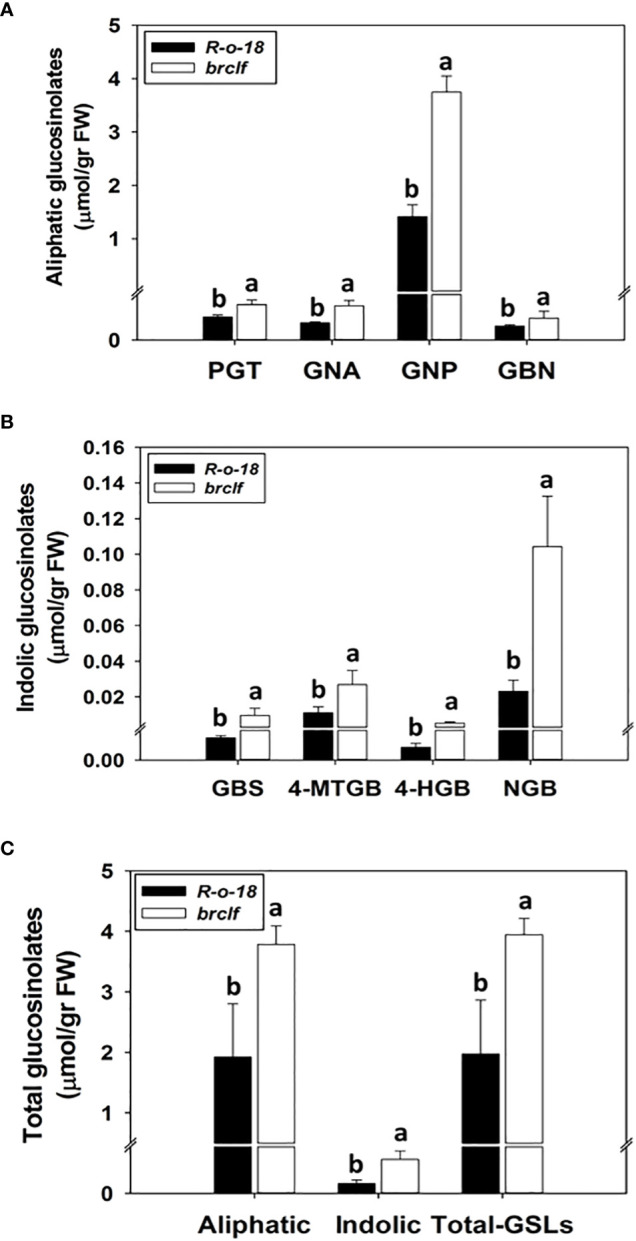
Comparison of the amounts of aliphatic and indolic GSL compounds between R-o-18 and *brclf* mutant. **(A)** Comparison of the amounts of four aliphatic GSL compounds between R-o-18 and *brclf* mutant. PGT: progoitrin, GNA: gluconapoleiferin, GNP: gluconapin, GBN: glucobrassicanapin. **(B)** Comparison of amounts of four indolic GSL compounds between R-o-18 and *brclf* mutant. GBS: glucobrassicin, 4-MTGB: 4-Methoxyglucobrassicin, 4-HGB: 4-hydroxyglucobrassicin, NGB: neoglucobrassicin. **(A–C)** One-way ANOVA with Tukey’s *post-hoc* test was applied to calculate statistical differences (p<0.05), and data were expressed as means ± standard deviation (SD). Statistical difference was indicated with different letters above each bar.

GSLs biosynthetic genes were reported to be positively modulated by a group of R2-R3 type MYB transcription factors like *BrMYB28*, *BrMYB29* for aliphatic GSLs and *BrMYB34*, *BrMYB51*, and *BrMYB122* for indolic GSLs (Gigolashvili et al., 2007; Hirai et al., 2007; Sønderby et al., 2007; Malitsky et al., 2008; Gigolashvili et al., 2009). When we compared the transcript levels of these MYB-domain TF genes, *BrMYB29* for aliphatic GSLs and *BrMYB34s* and *BrMYB122s* for indolic GSLs were substantially upregulated in *brclf* mutant in comparison to R-o-18 (**Figure 5**). It suggests that BrCLF play a negative role in the expression of *BrMYB* TFs genes regulating aliphatic and indolic GSL biosynthesis (**Figures 6**, **7**). To confirm that BrCLF is involved in the GSL metabolism, we quantified endogenous two major types of GSLs, aliphatic and indolic GSLs. We successfully detected four aliphatic GSL compounds (PGT, GNA, GNP, and GBN) and four indolic GSL compounds (GBS, 4-MTGB, 4-HGB, and NGB) using U-HPLC analysis (**Supplementary Figures S7A, B**). Compared to levels of R-o-18, *brclf* mutant had significantly increased amounts of both aliphatic and indolic GSLs (**Figure 8**). PCA analysis displayed that GSL compounds of R-o-18 and *brclf* were clustered in different groups but closely associated each other (**Supplementary Figure S7C**). Further, the biplot picture which generated from the PCA data pointed out that loss of BrCLF affected GSL metabolism (**Supplementary Figure S7D**). Altogether, these data indicated that BrCLF (and its PRC2 complex) suppresses genes involved in the stress signaling cascade and downstream stress-responsive GSL metabolism in *B. rapa* plants.

### H3K27me3 was enriched in “developmental genes,” “stress signaling” and stress-responsive “GSLs pathway genes”

**Figure 9:**
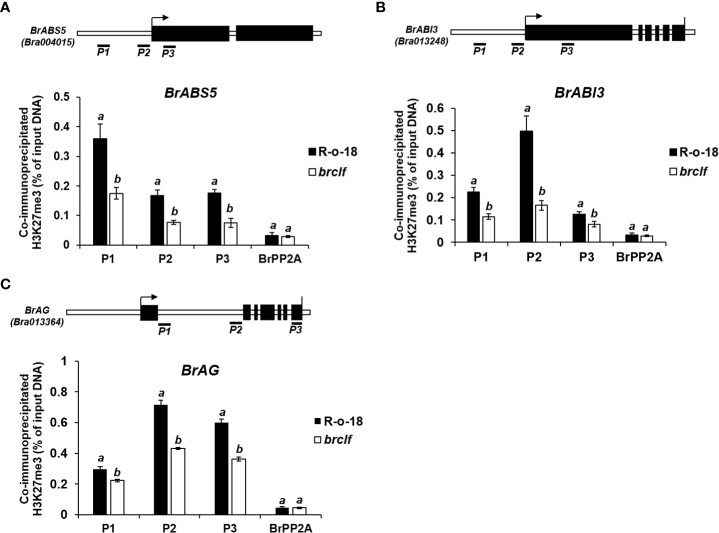
ChIP-qPCR analysis of 3 developmental genes between R-o-18 and *brclf* mutant. The enrichment level of H3K27me3 in developmental genes related to **(A)** leaf size (*BrABS5*-Bra004015) **(B)** seed dormancy (*BrABI3*-Bra013248), **(C)** floral organ identity (*BrAG*-Bra-013364) were calculated between R-o-18 and *brclf* mutant. All tested genes exhibited substantially reduced levels of H3K27me3 in *brclf* mutant compared with R-o-18 plant. Enrichment level was calculated as % of input DNA. *BrPP2A* (Bra012474) was used as a negative control (H3K27me3-unmarked gene) which was used as a housekeeping reference gene (Kim et al., 2022; **Supplementary Figure S13**). *BrFUS3* (Bra032953) was used as a positive control, H3K27me3-enriched gene (**Supplementary Figure S13**). Mean and standard deviation (SD) of three biological replicates were calculated and presented. Statistical analysis was performed with the one-way analysis of variance (ANOVA) and *post-hoc* Tukey’s test (p < 0.05).

*Arabidopsis* CLF-containing PRC2 complex play a suppressive role on target genes *via* deposit of H3K27me3 (Bouyer et al., 2011). Recently, genome-wide H3K27me3 profiles in leaf and flower tissues of R-o-18 inbred line (*B. rapa* ssp. *trilocularis*) were published (Payá-Milans et al., 2019). Thus, enrichment of H3K27me3 on developmental genes, stress signaling genes, and stress-responsive metabolic genes were analyzed in both “leaf” and “inflorescence”. In case of developmental genes, all tested 20 genes (100%) were markedly enriched with H3K27me3 in both leaf and flower tissues (**Supplementary Figure S8**). To validate this, we performed ChIP assay using H3K27me3 antibody between R-o-18 and *brclf* mutant. ChIP-qPCR analysis was performed on three developmental genes related to seed dormancy (*BrABI3*-Bra013248), leaf size (*BrABS5*-Bra004015), and floral organ identity (*BrAG*-Bra013364). All tested genes exhibited that enrichment of H3K27me3 was evidently compromised in the *brclf* mutant compared to R-o-18. (**Figure 9**). This result clearly explained why RNA-seq and qRT-PCR analyses of these developmental genes showed higher transcript levels in *brclf* mutants than R-o-18. It indicated that BrCLF regulates the expression and H3K27me3 marking of those genes, probably through direct deposition of the mark at these loci. A total of 13 “stress signaling” genes were also checked whether they have H3K27me3 enrichment on their genomic regions. 10 genes out of 13 (77%) stress-signaling genes were densely enriched with H3K27me3 (**Supplementary Figure S9**; **Supplementary Table S10**). Because stress signaling genes play a crucial role in the early stage of defense system, it is reasoned that BrCLF-containing PRC2 complex might contribute to suppress and maintain basal level expression of stress signal genes in normal condition prior to experience of challenging stress condition.

**Figure 10:**
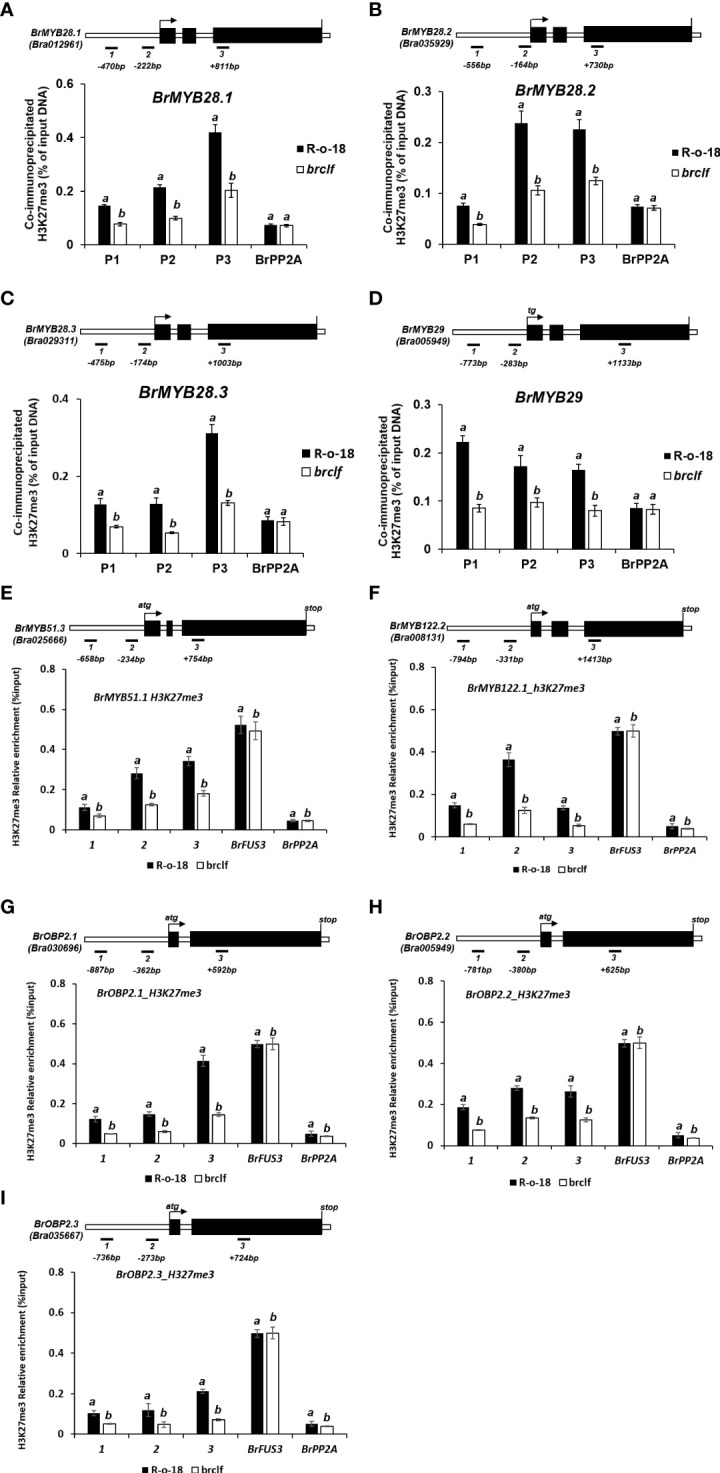
ChIP-qPCR analysis of 9 GSL TFs genes between R-o-18 and *brclf* mutant. The enrichment level of H3K27me3 in TF genes involved in the regulation of aliphatic GSL [*BrMYB28.1* **(A)**, *BrMYB28.2* **(B)**, *BrMYB28.3* **(C)**, and *BrMYB29* **(D)**] and Indolic GSL [*BrMYB51.3* **(E)**, *BrMYB122.2* **(F)**, *BrOBP2.1* **(G)**, *BrOBP2.2* **(H)**, *BrOBP2.3* **(I)**]. All tested genes exhibited substantially reduced levels of H3K27me3 in *brclf* mutant compared with R-o-18 plant. Enrichment level was calculated as % of input DNA. *BrPP2A* (Bra012474) was used as a negative control (H3K27me3-unmarked gene) which was used as a housekeeping reference gene (Kim et al., 2022; **Supplementary Figure S13**). *BrFUS3* (Bra032953) was used as a positive control, H3K27me3-enriched gene (**Supplementary Figure S13**). Mean and standard deviation (SD) of three biological replicates were calculated and presented. Statistical analysis was performed with the one-way analysis of variance (ANOVA) and *post-hoc* Tukey’s test (p < 0.05).

In case of stress-responsive metabolites, aliphatic and indolic GSLs, we checked the presence of H3K27me3 in 104 pathway genes, 15 TF genes (4 genes for aliphatic and 11 genes for indolic pathway), and 89 GSL biosynthesis genes comprising 59 aliphatic and 30 indolic pathway genes. Interestingly, fourteen genes out of 15 TF genes (93%) except *BrMYB34.3* were enriched with H3K27me3 in either leaf or flower (**Supplementary Figure S10**; **Supplementary Table S11**). To quantify relative enrichment of H3K27me3 in the GSL TFs, ChIP-qPCR analysis was applied for both of aliphatic and indolic GSL TFs which includes three TFs involved in aliphatic GSL (*BrMYB28.1, BrMYB28.2*, and *BrMYB28.3*) and five TFs for indolic GSL (*Br BrMYB51.3, BrMYB122.1, BrOBP2.1, BrOBP2.2, BrOBP2.3*). The result showed that ‘*brclf*’ contained low level of H3K27me3 compared to levels of R-o-18 (**Figure 10**). Thus, it is confirmed that BrCLF, a component of *B. rapa* PRC2 complex is required for H3K27me3 enrichment for the suppression of target genes.

We also checked the presence of H3K27me3 in 59 aliphatic and 30 indolic glucosinolates biosynthesis genes. Of the total 59 aliphatic pathway genes, 32 genes (54%) were enriched with H3K27me3 in their genomic regions in either leaf or flower tissue (**Supplementary Figure S11**; **Table S11**). In case of 30 indolic GSL pathway genes, 18 (60%) genes were accumulated with H3K27me3 in their genomic regions in either leaf or flower tissue (**Supplementary Figure S12**; **Table S11**). These results indicated that many TF genes and biosynthetic genes (to a lesser degree) related to aliphatic and indolic GSLs were under the epigenetic control mediated by BrCLF-containing PRC2 complex in *B. rapa*. Thus, our study combining RNA-seq, H3K27me3 ChIP-qPCR dataset, and metabolic analysis between R-o-18 and *brclf* demonstrated that BrCLF-containing PRC2 complex modulate expression of key developmental genes (i.e., floral transition, seed dormancy, floral organ development) and genes related to stress signaling and stress-responsive secondary metabolisms (aliphatic and indolic glucosinolates) in *B. rapa* plants.

## Discussion

Plants are constantly exposed to biotic (e.g., insect, fungi, and bacterial attacks) and abiotic stresses (e.g., drought, heat, salinity) during their lifetime. As sessile organisms with limited resources, they need to execute growth-defense tradeoffs, which are well-balanced optimization between growth and defense programs (Huot et al., 2014). Both programs are costly and critical for their survival, adaptation, and successful reproduction. Upon challenging stress, plants rapidly respond and use a defense mode by initiating a complex network of signaling cascades, which trigger genome-wide transcriptomic changes to build a multi-layered defense system against threatening stressors. However, molecular mechanisms underlying growth and defense tradeoffs remain poorly elucidated. In this study, we found that genes involved in crucial developmental transitions were highly enriched with an H3K27me3 histone mark, highlighting the importance of epigenetic control in developmental programs in *B. rapa* (**Figure 9**; **Supplementary Figure S8**). In addition, we revealed that high proportions of stress signaling genes and glucosinolate (aliphatic and indolic) biosynthesis genes were densely enriched with H3K27me3, indicating that these stress responsive genes were suppressed by a *BrCLF*-containing PRC2 complex in *B. rapa* (**Figure 10**; **Supplementary Figures S9**–**S12**). It is worthy to note that recent transcriptomic analysis of *Arabidopsis* found that CLF regulate the genes involved in the aliphatic and indolic GSLs pathways including side-chain elongation, core structure formation, and secondary modification steps of *Arabidopsis* model plant (Tyler et al., 2019). Thus, it is likely that CLF and BrCLF-mediated H3K27me3 might commonly suppress stress-responsive genes in the absence of challenging stress to benefit plant developmental growth and simultaneously save unessential costs on defense programs.

PRCs play an essential role in diverse developmental programs in many eukaryotes (Margueron and Reinberg, 2011). In an *Arabidopsis* model plant, CLF and SWN, which are PRC2 components, encode H3K27me3 methyltransferases. Thus, CLF/SWN-containing PRC2 catalyzes the trimethylation of H3K27, which is a histone mark for gene inactivation (Kim and Sung, 2014). Numerous genes related to developmental transition processes, such as seed germination, juvenile-to-adult phase transition, leaf and floral organ development, and floral transition, are enriched with H3K27me3 in *Arabidopsis* (Bouyer et al., 2011). In addition to the role of PRCs in developmental gene regulation, the functional role of PRCs in the regulation of stress-related pathway genes in *Arabidopsis* model plant has been described. For instance, a recent study reported that *Arabidopsis* CLF, a PRC2 component, is positively required for the expression of *ORA59*, a stress-responsive AP2/ERF transcription factor. The reduced *ORA59* expression in a *clf* mutant results in at least a partial defect in leaf immunity because of the low expression of defense-related genes such as *PDF1.2a* (Kosaka et al., 2020; Singkaravanit-Ogawa et al., 2021). On the contrary, in our study using *B. rapa*, *ORA59* was upregulated in the *brclf* mutant compared with that in the R-o-18 wild type; furthermore, H3K27me3 was densely enriched in the *ORA59* chromatin region (**Figure 4B**; **Supplementary Figure S9**). These data indicated that BrCLF played a negative role in the *ORA59* expression by depositing H3K27me3 in *B. rapa*. Therefore, even though *Arabidopsis* and *B. rapa* belong to Brassicaceae, *B. rapa* homologs probably evolved to acquire a different and divergent role from *Arabidopsis* homologs.

LHP1/TFL2 is a PRC1 component that physically recognizes and binds to H3K27me3; then, it subsequently carries other PRC1 components to stably silence target genes (Turck et al., 2007; Veluchamy et al., 2016). LHP1 is required for the repression of stress signaling genes, such as *MYC2* and NAC domain protein, *ANAC55* (*NAC DOMAIN CONTAINING PROTEIN 55*), which are downstream target genes of *ORA59* and involved in the jasmonate (JA) and ethylene (ET) signaling pathway of immunity in *Arabidopsis* (Ramirez-Prado et al., 2019). H3K27me3 and LHP1 are highly enriched in the *MYC2* and *ANAC55* region, indicating that LHP1-containing PRC1 suppresses this defense signaling factors in *Arabidopsis*. Consequently, *MYC2* and *ANAC55* are upregulated in the loss-of-function LHP1 because of the reduced enrichment of the H3K27me3 mark. In the *lhp1* mutant, aphid resistance, drought stress tolerance, and abscisic acid (ABA) sensitivity are increased. In this study, we identified one *B. rapa MYC2* homolog (named *BrMYC2*) and two *B. rapa ANAC55* homologs (named *BrANAC55.1* and *BrANAC55.2*) from the *B. rapa* genome (**Supplementary Table S7**). These homologs were upregulated in *brclf* mutant compared with R-o-18 (**Figure 4**). Regarding the enrichment of H3K27me3, *BrANAC55.2* (Bra021113) was evidently enriched with H3K27me3, indicating that it is the direct target of BrCLF-containing PRC2 complex in *B. rapa*. Meanwhile, *BrMYC2* (Bra010178) and *BrANAC55.1* (Bra027238) were not enriched with H3K27me3 indicating that they might be not the direct target of PRC2. Nevertheless, their expressions were higher in the *brclf* mutant than R-o-18, possibly because of the upregulated upstream transcription factors like *BrORA59*. It need further investigation that BrORA59 is direct positive regulator of *BrMYC2* and *BrANAC55s* mediated defense signaling pathway in *B. rapa*.

In *Arabidopsis*, MYC2 activates glucosinolate biosynthesis in signaling mediated by a defense hormone, namely, jasmonic acid (JA) (Pangesti et al., 2016). *B. rapa* has only a single homolog of *MYC2* in *B. rapa*, (*BrMYC2*). This study showed that *BrMYC2* is not enriched with H3K27me3, indicating that *BrMYC2* might not be the direct target of PRC2 (**Supplementary Table S10**). Nonetheless, the *BrMYC2* expression was higher in *brclf* than in R-o-18 (**Figure 4**; **Supplementary Table S7**). Therefore, the upstream regulators of *BrMYC2* in the *brclf* mutant were influenced, affecting *BrMYC2* expression. Alternatively, JA-related biosynthesis genes were upregulated in *BrCLF* mutation; as a result, the increased amount of endogenous JA enhanced the *BrMYC2* expression in the *brclf* mutant.

In *Arabidopsis*, CLF and its closest paralog, SWN, functioned as H3K27me3 methyltransferase in genes related to many facets of developmental programs (**Supplementary Figure S1B**). The null mutant of *CLF* exhibited serious developmental defects, such as small and curly leaves, deformed floral organs, and early flowering, indicating that CLF are implicated in *Arabidopsis* developmental programs. Meanwhile, *swn*, loss-of-function *SWN*, does not exhibit an abnormal phenotype compared with wild-type plants (Shu et al., 2020). Double mutants of *CLF* and *SWN* display more severe developmental defects than the single mutant of *CLF* does, indicating that CLF and SWN play at least partly redundant roles in developmental programs in *Arabidopsis*. We noticed that the percentage of upregulated genes in *brclf* (35 genes/104 total, 34%) was lower than that of the GSL pathway genes enriched with H3K27me3 (64 genes/104 total, 62%) (**Supplementary Tables S8**, **S11**). Regarding this discrepancy, we reasoned that some genes not affected in *brclf* might be redundantly suppressed by BrSWN; thus, they were enriched with H3K27me3, but their expression was not significantly affected. Therefore, the development of a *BrSWN* mutant might further clarify the functional redundancy between *BrCLF* and *BrSWN* in *B. rapa*.

MEA, another paralog of CLF and SWN, also encodes an H3K27me3 methyltransferase and is uniquely expressed in the early embryo of *Arabidopsis* (Arnaud and Feil, 2006). It serves as a catalytic unit of PRC2 to negatively regulate seed development in the absence of fertilization (Haun et al., 2007). Even though MEA acts as an H3K27me3 transferase in genomic imprinting for proper embryo development, it likely plays another role in plant immune system (Roy et al., 2018). Its transcription is triggered when plants are inoculated with pathogens such as *Pseudomonas syringae* pv. *Tomato* (*Pst*); this process also has a positive role in *Pst-AvrRpt2* carrier-mediated pathogenesis (Roy et al., 2018). An induced MEA acts to suppress the plant defense gene *RESISTANCE TO P. SYRINGAE 2* (*RPS2*), thus weakening plant immunity against pathogen attack. Therefore, the functional role of MEA and CLF in stress response might be divergently evolved in *Arabidopsis* although they have a similar repressive role in developmental genes by depositing H3K27me3. In this study, *B. rapa CLF* homolog was determined, and the importance of *BrCLF* in the transcriptional regulation of genes related to development and stress-responsive genes (i.e., glucosinolates); however, further studies on other *BrCLF* paralogs, *BrSWN*, *BrMEA.a*, and *BrMEA.b* should be conducted to clarify functional similarity and diversity in response to different types of abiotic and biotic stresses in *B. rapa*.

Gene activation and repression are highly correlated with the contents of histone modifications in an individual genomic region. For example, active histone marks such as H3K4me3, H3K36me3, and H3ac are correlated with gene activation, whereas H3K27me3, H3K9me3, and deacetylated histone H3/H4 are highly related to gene repression (Kouzarides, 2007; Ramirez-Prado et al., 2018). In this context, stress signaling and stress-responsive metabolic genes might undergo dynamic histone modification before and after stress. However, further studies have yet to clarify whether the activation of stress-responsive genes upon exposure to stress requires the removal of accumulated H3K27me3 in their chromatin regions prior to activation; studies have also yet to determine if the H3K27me3 mark is constantly retained and if the activation of these stress-responsive genes can be independently achieved by stress-triggered transcriptional activators (i.e., *MYC2*). One recent study using *Arabidopsis* investigated the genome-wide profiles of different histone modifications before and after wounding stress (Rymen et al., 2019). This study suggested that enriched profiles of H3K27me3 were not considerably affected; instead, histone acetylation or active marks evidently accumulated in stress-related genes. To our best knowledge, however, studies have yet to determine whether stress signaling and stress-responsive metabolic genes in *B. rapa* undergoes dynamic histone modifications upon exposure to stress, similar to the case of *Arabidopsis*. Therefore, further genome-wide epigenetic profiling of different (active and repressive) histone modifications might provide further insights into the epigenetic regulation of stress response in *B. rapa*.

## Data availability statement

The datasets presented in this study can be found in online repositories. The names of the repository/repositories and accession number(s) can be found below: https://www.ncbi.nlm.nih.gov/, GSE214271.

## Author contributions

D-HK planned this study. AN and SK prepared all materials and performed genetic and molecular experiments. AN, SK, and D-HK performed bioinformatic analysis. AN and SK performed HPLC analysis. and D-HK wrote the manuscript. All authors contributed to the article and approved the submitted version.

## Supplementary material

Supplementary Figure 1 Identification of loss of function mutant of *BrCLF*, *brclf*. **(A)** Multiple alignment of amino acid sequences of *Arabidopsis* CLF (AtCLF) clade and *B. rapa* homologous genes, (named *BrCLF*, *BrSWN*, *BrMEA.a*, and *BrMEA.b*). Catalytic SET domain for H3K27 (H3K27me3) trimethylation was indicated with a green box. **(B)** Phylogenic analysis of *Arabidopsis* CLF (AtCLF) clade and *B. rapa* homologous proteins (BrCLF, BrSWN, BrMEA.a, and BrMEA.b). AtCLF has a single *B. rapa* homolog, BrCLF (Bra032169, indicated with red letters). **(C)** Sanger sequencing validation of point mutation in 11th exon of *BrCLF* coding sequence in *brclf* mutant. Nucleotide at 1,843bp from the start codon of *BrCLF* was converted from C to T which generate a premature stop codon in *brclf* mutant. **(D)** Comparison of the full amino acid sequence of BrCLF of R-o-18 and *brclf* mutant. Glutamine (Gln) residue at 615^th^ was converted to a stop codon (indicated with asterisk), generating the truncated form of BrCLF missing the H3K27me3 catalytic SET domain in the C-terminal region.

Supplementary Figure 2 Measurement of phenotypic differences between R-o-18 wild type and *brclf* mutant at **(A)** vegetative and **(B)** reproductive stages. **(A)** Measurement of hypocotyl length, leaf length, leaf width, and rosette diameter at 4-week-old seedling R-o-18 and *brclf*. **(B)** Measurement of plant height, petal length, number of siliques per plant, bud length, pistil length and number of seeds per a pod between R-o-18 and *brclf*. **(A–B)** One-way ANOVA with Tukey’s *post-hoc* test was applied to calculate statistical differences (p < 0.05), and data were expressed as means ± standard deviation (SD). Statistical difference was indicated with different letters above each bar. Phenotypic measurement for vegetative organs including hypocotyl, leaf, rosette, and plant height was analyzed from 8 individual plants (n=8). Meanwhile, the measurement of petal, siliques, bud, pistil, and number of seeds were obtained from 5 individual plants (n=5).

Supplementary Figure 3 Quality check of RNA-seq reads. **(A)** Correlation heatmap analysis showing distinct clustering between R-o-18 and *brclf* samples. Three biological replicates of R-o-18 and *brclf* were used for RNA-seq library construction. **(B)** MA plots of differentially expressed genes between R-o-18 and *brclf*. X-axis indicates the average expression and logCPM (log_2_ transformed expression value), and the Y-axis indicates the value of log_2_-transformed fold change. Red dots indicate differentially expressed genes from R-o-18 and *brclf* samples. Black dots indicate non-DEGs.

Supplementary Figure 4 qRT-PCR analysis of 12 developmental genes between R-o-18 and *brclf* mutant. Transcript levels of developmental genes related to **(A)** seed dormancy, **(B)** leaf size, **(C)** floral organ identity, and **(D)** floral transition were substantially upregulated in *brclf* mutant compared with those in the wild type R-o-18 plant. Total RNAs extracted from 2-week-old seedlings were used for qRT-PCR analysis. Mean and standard deviation (SD) of three biological replicates were calculated and presented. One-way ANOVA analysis with Tukey *post-hoc* test was applied to determine statistical significance (p < 0.05).

Supplementary Figure 5 Venn diagram between the list of *B. rapa* 5,122 stress-related genes and 1,844 upregulated genes in brclf compared to R-o-18. Total 301 stress-related genes were found in the 1,844-upregulated genes in the *brclf* mutant. VENNY (ver. 2.1) was used to generate a Venn diagram ().

Supplementary Figure 6 Quantification of transcript levels of oxidative stress-related genes between R-o-18 and *brclf via* qRT-PCR. Mean and standard deviation (SD) of three biological replicates were calculated and presented. One-way ANOVA analysis with Tukey’s *post-hoc* test was applied to determine statistical significance (p < 0.05).

Supplementary Figure 7 Profiles of glucosinolate (GSL) compounds detected from R-o-18 and *brclf* seedling plants through HPLC analysis. HPLC analysis detected total 8 GSL compounds (4 aliphatic GSL compounds and 4 indolic GSL compounds) were detected from both **(A)** R-o-18 and **(B)** *brclf*. Principal component analysis (PCA) scores **(C)** and biplot **(D)** of GSL distribution between R-o-18 and *brclf*. Green dots and red triangle denote R-o-18 and *brclf*, respectively. Principal component analysis (PCA) and biplot for the GSL content of R-o-18 and *brclf* was performed using Metaboanalyst 5.0 (). PGT: progoitrin, GNA: gluconapoleiferin, GNP: gluconapin, GBN: glucobrassicanapin. GBS: glucobrassicin, 4-MTGB: 4-Methoxyglucobrassicin, 4-HGB: 4-hydroxyglucobrassicin, NGB: neoglucobrassicin. Three biological replicates for R-o-18 and *brclf* were used for HPLC analysis.

Supplementary Figure 8 Integrative genome browser (IGV) illustration showing enrichment of H327me3 on 20 developmental transitions in leaf and flower tissues. Normalized enrichment of H3K27me3 on 20 developmental genes related to **(A)** seed dormancy, **(B)** floral organ development, **(C)** leaf development, and **(D)** floral transition in *B. rapa* from leaf and flower tissues compared with the normalized input signal levels. All the 20 tested developmental genes were enriched with H3K27me3. Black tracks indicate the normalized input DNA intensity; green and orange tracks refer to the normalized α-H3K27me3-immunoprecipitated DNA intensity of leaf and flower tissues, respectively. ChIP-seq read intensity in the Y-axis is indicated with a bracket on the left upper part of each track.

Supplementary Figure 9 Integrative genome browser (IGV) illustration showing enrichment of H327me3 on stress signaling related genes in leaf and flower tissues. Normalized enrichment of H3K27me3 histone mark on 13 oxidative stress signaling genes from leaf and flower tissues compared with normalized input signal levels. Of the 13 stress signaling genes, 10 (77%) were enriched with H3K27me3. Black tracks indicate normalized input DNA intensity; green and orange tracks indicate the normalized α-H3K27me3-immunoprecipitated DNA intensity of leaf and flower tissues, respectively. ChIP-seq read intensity in the Y-axis is indicated with a bracket on the left upper part of each track.

Supplementary Figure 10 IGV illustration showing H327me3 enrichment on transcription factor genes controlling aliphatic and indolic GSL biosynthesis in leaf and flower tissues. **(A)** Normalized enrichment of H3K27me3 histone mark on 4 *BrMYB* TF genes controlling aliphatic GSL biosynthesis from both leaf and flower tissues compared with levels of the normalized input signal. **(B)** Normalized enrichment of H3K27me3 histone mark on 11 TF genes controlling indolic GSL biosynthesis from both leaf and flower tissues compared with levels of the normalized input signal. **(A–B)** Black color tracks indicate normalized input DNA intensity, green and orange color tracks indicate the normalized α-H3K27me3-immunoprecipitated DNA intensity of leaf and flower tissues, respectively. ChIP-seq read intensity of Y-axis is indicated with bracket on left upper part of each track.

Supplementary Figure 11 Integrative genome browser (IGV) illustration showing H327me3 enrichment in 59 aliphatic glucosinolate (GSL) biosynthesis genes in leaf and flower tissues. Normalized enrichment of H3K27me3 histone mark in genes related to the aliphatic GSL biosynthesis from leaf and flower tissues compared with levels of the normalized input signal. **(A)** Enrichment of H3K27me3 in 19 genes related to side-chain elongation process of aliphatic GSLs pathway. **(B)** Enrichment of H3K27me3 in 17 genes related to core structure formation process of aliphatic GSLs pathway. **(C)** Enrichment of H3K27me3 in 16 genes related to secondary modification process of aliphatic GSL pathway. **(A–C)** Black tracks indicate normalized input DNA intensity; green and orange tracks indicate the normalized α-H3K27me3-immunoprecipitated DNA intensity of leaf and flower tissues, respectively. ChIP-seq read intensity of Y-axis is indicated with a bracket on the left upper part of each track.

Supplementary Figure 12 Integrative genome browser (IGV) illustration showing H327me3 enrichment in 40 indolic glucosinolate (GSL) biosynthesis genes in leaf and flower tissues. Normalized enrichment of H3K27me3 histone mark in genes related to indolic GSL biosynthesis from both leaf and flower tissues compared with levels of the normalized input signal. **(A)** Enrichment of H3K27me3 in 10 genes related to core structure formation process of indolic GSLs pathway. **(B)** Enrichment of H3K27me3 in 12 genes related to secondary modification process of indolic GSLs pathway. **(C)** Enrichment of H3K27me3 on 7 genes commonly involved in both aliphatic and indolic GSL pathways. **(A–C)** Black tracks indicate normalized input DNA intensity; green and orange tracks indicate the normalized α-H3K27me3-immunoprecipitated DNA intensity of leaf and flower tissues, respectively. ChIP-seq read intensity of Y-axis is indicated with a bracket on the left upper part of each track.

Supplementary Figure 13 Verification of H3K27me3-unmarked *BrPP2A* and H3K27me3-marked *BrFUS3* in *B. rapa*. **(A)** IGV genome browser visualization showing the transcript levels of *BrPP2A* (Bra012474) between R-o-18 and *brclf* mutant. Transcription of *BrPP2A* was not affected in *brclf* in comparison to level of R-o-18. Three biological replicates from RNA-seq read counts were indicated with black color (R-o-18) and brown color (*brclf*). **(B)** IGV genome browser visualization showing the enriched level of H3K27me3 at the genomic locus of *BrPP2A* (Bra012474) in either leaf or flower tissue in comparison to input DNA. Upper diagram presents a primer location used for ChIP-qPCR analysis. A housekeeping reference gene, *BrPP2A* was not enriched with H3K27me3. ChIP-seq read intensity of Y-axis is indicated with a bracket on the left upper part of each track. **(C)** Result of ChIP-qPCR on H3K27me3 enrichment between R-o-18 and *brclf* mutant at *BrFUS3* (Bra032953) genomic locus. Genomic region of BrFUS3 was enriched with H3K27me3 in comparison to input DNA. Upper diagram presents a primer location used for ChIP-qPCR analysis. One-way ANOVA analysis with Tukey’s *post-hoc* test was applied to determine statistical significance (p < 0.05).
