# Supplementary figures and images for: Transcriptomic and epigenomic analyses revealed that polycomb repressive complex 2 regulates not only developmental but also stress responsive metabolism in Brassica rapa
Source: Front Plant Sci. 2023 Feb 20;14:1079218. doi: 10.3389/fpls.2023.1079218 (PMC9986605; doi:10.3389/fpls.2023.1079218)

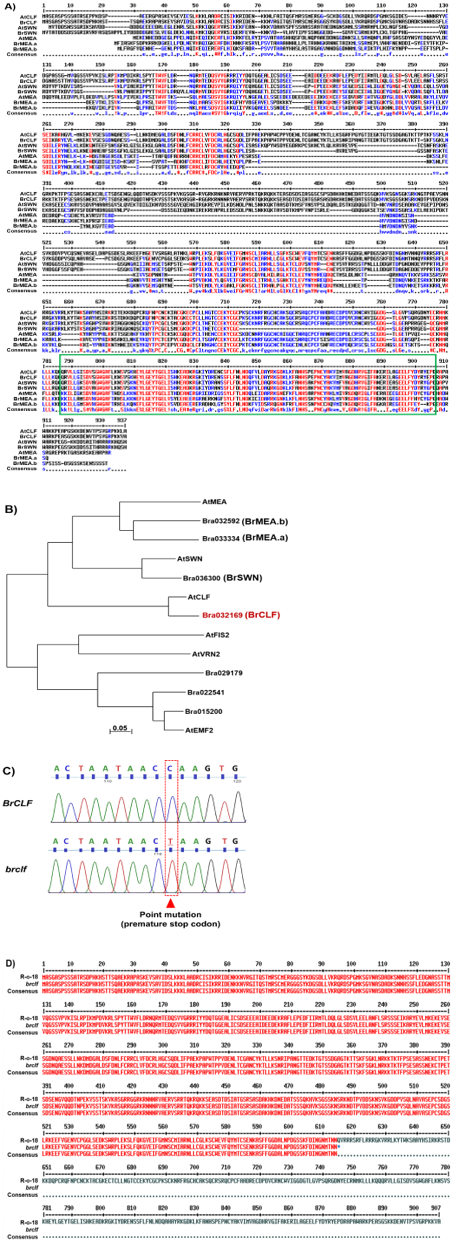

Supplement: Supplementary Figure 1 — Identification of loss of function mutant of BrCLF, brclf. (A) Multiple alignment of amino acid sequences of Arabidopsis CLF (AtCLF) clade and B. rapa homologous genes, (named BrCLF, BrSWN, BrMEA.a, and BrMEA.b). Catalytic SET domain for H3K27 (H3K27me3) trimethylation was indicated with a green box. (B) Phylogenic analysis of Arabidopsis CLF (AtCLF) clade and B. rapa homologous proteins (BrCLF, BrSWN, BrMEA.a, and BrMEA.b). AtCLF has a single B. rapa homolog, BrCLF (Bra032169, indicated with red letters). (C) Sanger sequencing validation of point mutation in 11th exon of BrCLF coding sequence in brclf mutant. Nucleotide at 1,843bp from the start codon of BrCLF was converted from C to T which generate a premature stop codon in brclf mutant. (D) Comparison of the full amino acid sequence of BrCLF of R-o-18 and brclf mutant. Glutamine (Gln) residue at 615th was converted to a stop codon (indicated with asterisk), generating the truncated form of BrCLF missing the H3K27me3 catalytic SET domain in the C-terminal region. [file DataSheet_1.zip › (rev)Supp Fig. S1.tif]

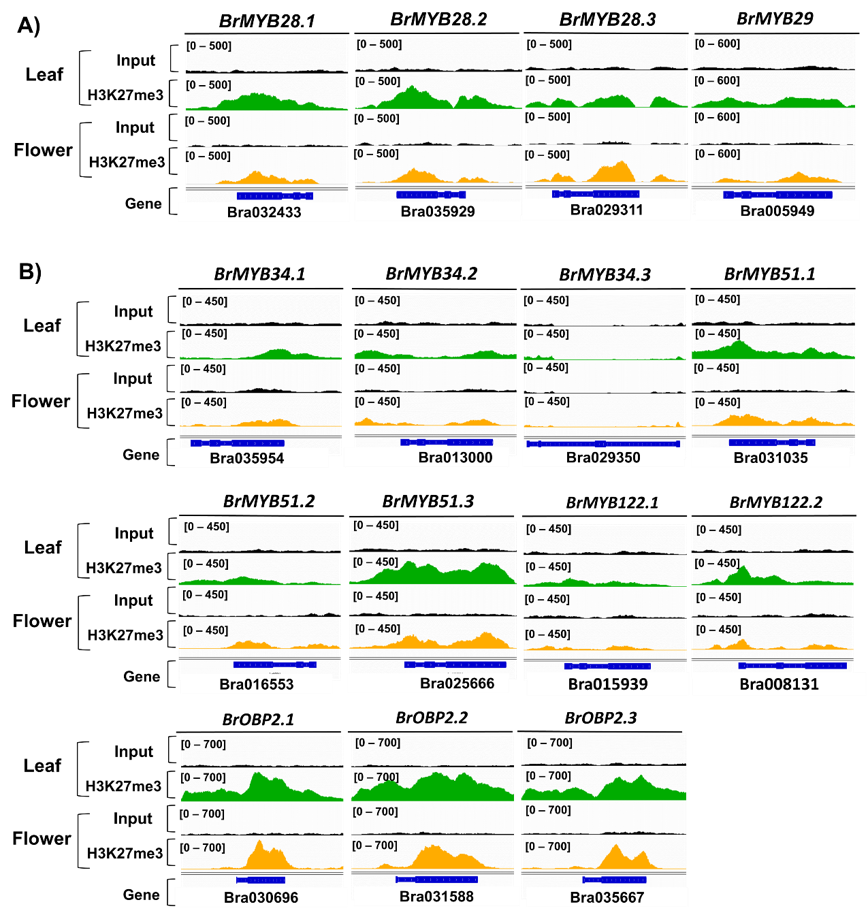

Supplement: Supplementary Figure 1 — Identification of loss of function mutant of BrCLF, brclf. (A) Multiple alignment of amino acid sequences of Arabidopsis CLF (AtCLF) clade and B. rapa homologous genes, (named BrCLF, BrSWN, BrMEA.a, and BrMEA.b). Catalytic SET domain for H3K27 (H3K27me3) trimethylation was indicated with a green box. (B) Phylogenic analysis of Arabidopsis CLF (AtCLF) clade and B. rapa homologous proteins (BrCLF, BrSWN, BrMEA.a, and BrMEA.b). AtCLF has a single B. rapa homolog, BrCLF (Bra032169, indicated with red letters). (C) Sanger sequencing validation of point mutation in 11th exon of BrCLF coding sequence in brclf mutant. Nucleotide at 1,843bp from the start codon of BrCLF was converted from C to T which generate a premature stop codon in brclf mutant. (D) Comparison of the full amino acid sequence of BrCLF of R-o-18 and brclf mutant. Glutamine (Gln) residue at 615th was converted to a stop codon (indicated with asterisk), generating the truncated form of BrCLF missing the H3K27me3 catalytic SET domain in the C-terminal region. [file DataSheet_1.zip › (rev)Supp Fig. S10.tif]

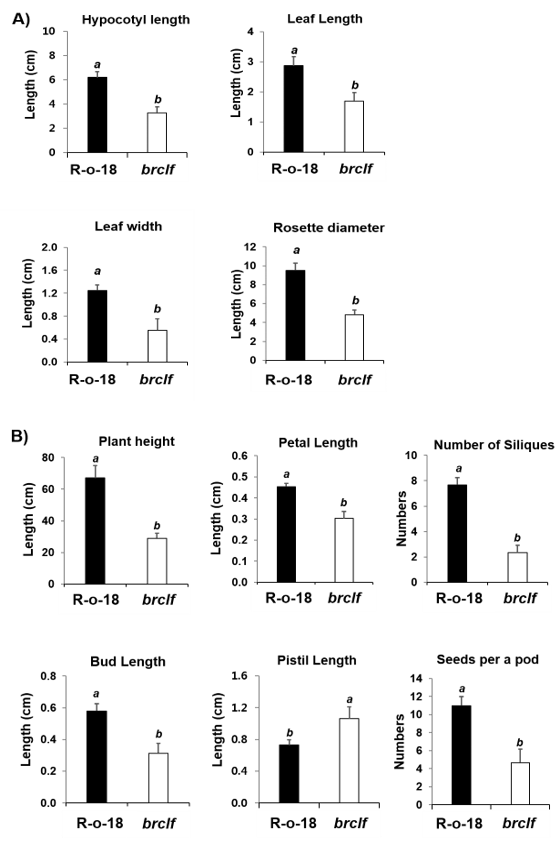

Supplement: Supplementary Figure 1 — Identification of loss of function mutant of BrCLF, brclf. (A) Multiple alignment of amino acid sequences of Arabidopsis CLF (AtCLF) clade and B. rapa homologous genes, (named BrCLF, BrSWN, BrMEA.a, and BrMEA.b). Catalytic SET domain for H3K27 (H3K27me3) trimethylation was indicated with a green box. (B) Phylogenic analysis of Arabidopsis CLF (AtCLF) clade and B. rapa homologous proteins (BrCLF, BrSWN, BrMEA.a, and BrMEA.b). AtCLF has a single B. rapa homolog, BrCLF (Bra032169, indicated with red letters). (C) Sanger sequencing validation of point mutation in 11th exon of BrCLF coding sequence in brclf mutant. Nucleotide at 1,843bp from the start codon of BrCLF was converted from C to T which generate a premature stop codon in brclf mutant. (D) Comparison of the full amino acid sequence of BrCLF of R-o-18 and brclf mutant. Glutamine (Gln) residue at 615th was converted to a stop codon (indicated with asterisk), generating the truncated form of BrCLF missing the H3K27me3 catalytic SET domain in the C-terminal region. [file DataSheet_1.zip › (rev)Supp Fig. S2.tif]

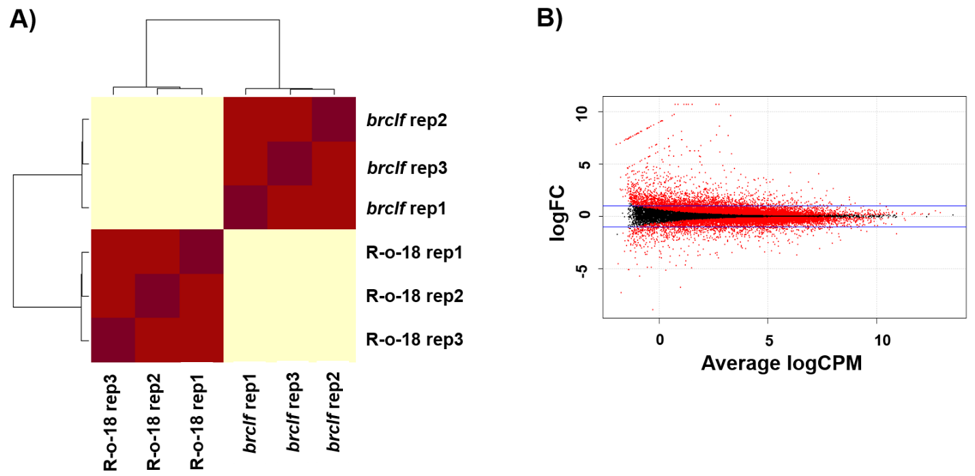

Supplement: Supplementary Figure 1 — Identification of loss of function mutant of BrCLF, brclf. (A) Multiple alignment of amino acid sequences of Arabidopsis CLF (AtCLF) clade and B. rapa homologous genes, (named BrCLF, BrSWN, BrMEA.a, and BrMEA.b). Catalytic SET domain for H3K27 (H3K27me3) trimethylation was indicated with a green box. (B) Phylogenic analysis of Arabidopsis CLF (AtCLF) clade and B. rapa homologous proteins (BrCLF, BrSWN, BrMEA.a, and BrMEA.b). AtCLF has a single B. rapa homolog, BrCLF (Bra032169, indicated with red letters). (C) Sanger sequencing validation of point mutation in 11th exon of BrCLF coding sequence in brclf mutant. Nucleotide at 1,843bp from the start codon of BrCLF was converted from C to T which generate a premature stop codon in brclf mutant. (D) Comparison of the full amino acid sequence of BrCLF of R-o-18 and brclf mutant. Glutamine (Gln) residue at 615th was converted to a stop codon (indicated with asterisk), generating the truncated form of BrCLF missing the H3K27me3 catalytic SET domain in the C-terminal region. [file DataSheet_1.zip › (rev)Supp Fig. S3.tif]

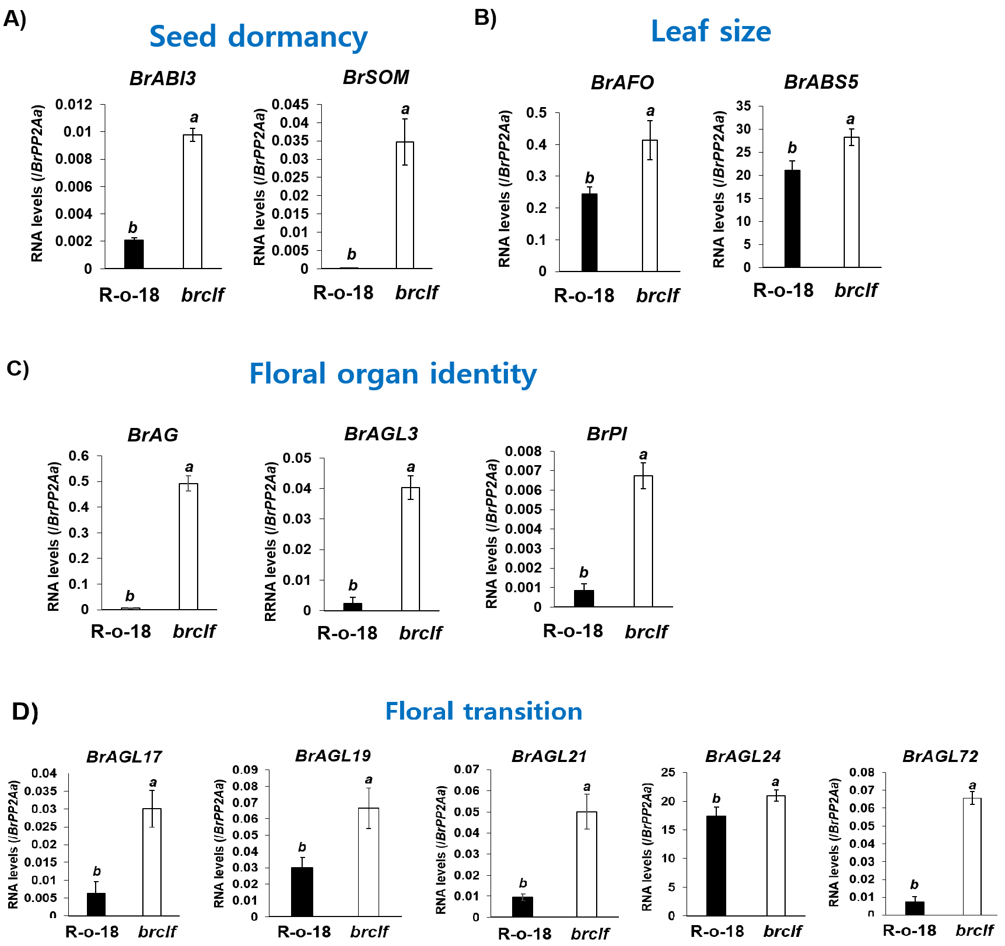

Supplement: Supplementary Figure 1 — Identification of loss of function mutant of BrCLF, brclf. (A) Multiple alignment of amino acid sequences of Arabidopsis CLF (AtCLF) clade and B. rapa homologous genes, (named BrCLF, BrSWN, BrMEA.a, and BrMEA.b). Catalytic SET domain for H3K27 (H3K27me3) trimethylation was indicated with a green box. (B) Phylogenic analysis of Arabidopsis CLF (AtCLF) clade and B. rapa homologous proteins (BrCLF, BrSWN, BrMEA.a, and BrMEA.b). AtCLF has a single B. rapa homolog, BrCLF (Bra032169, indicated with red letters). (C) Sanger sequencing validation of point mutation in 11th exon of BrCLF coding sequence in brclf mutant. Nucleotide at 1,843bp from the start codon of BrCLF was converted from C to T which generate a premature stop codon in brclf mutant. (D) Comparison of the full amino acid sequence of BrCLF of R-o-18 and brclf mutant. Glutamine (Gln) residue at 615th was converted to a stop codon (indicated with asterisk), generating the truncated form of BrCLF missing the H3K27me3 catalytic SET domain in the C-terminal region. [file DataSheet_1.zip › (rev)Supp Fig. S4.tif]

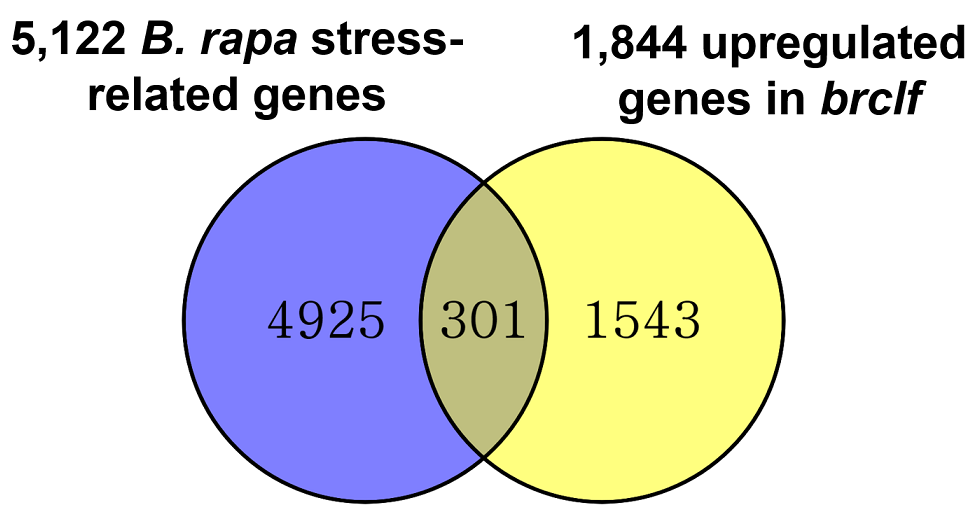

Supplement: Supplementary Figure 1 — Identification of loss of function mutant of BrCLF, brclf. (A) Multiple alignment of amino acid sequences of Arabidopsis CLF (AtCLF) clade and B. rapa homologous genes, (named BrCLF, BrSWN, BrMEA.a, and BrMEA.b). Catalytic SET domain for H3K27 (H3K27me3) trimethylation was indicated with a green box. (B) Phylogenic analysis of Arabidopsis CLF (AtCLF) clade and B. rapa homologous proteins (BrCLF, BrSWN, BrMEA.a, and BrMEA.b). AtCLF has a single B. rapa homolog, BrCLF (Bra032169, indicated with red letters). (C) Sanger sequencing validation of point mutation in 11th exon of BrCLF coding sequence in brclf mutant. Nucleotide at 1,843bp from the start codon of BrCLF was converted from C to T which generate a premature stop codon in brclf mutant. (D) Comparison of the full amino acid sequence of BrCLF of R-o-18 and brclf mutant. Glutamine (Gln) residue at 615th was converted to a stop codon (indicated with asterisk), generating the truncated form of BrCLF missing the H3K27me3 catalytic SET domain in the C-terminal region. [file DataSheet_1.zip › (rev)Supp Fig. S5.tif]

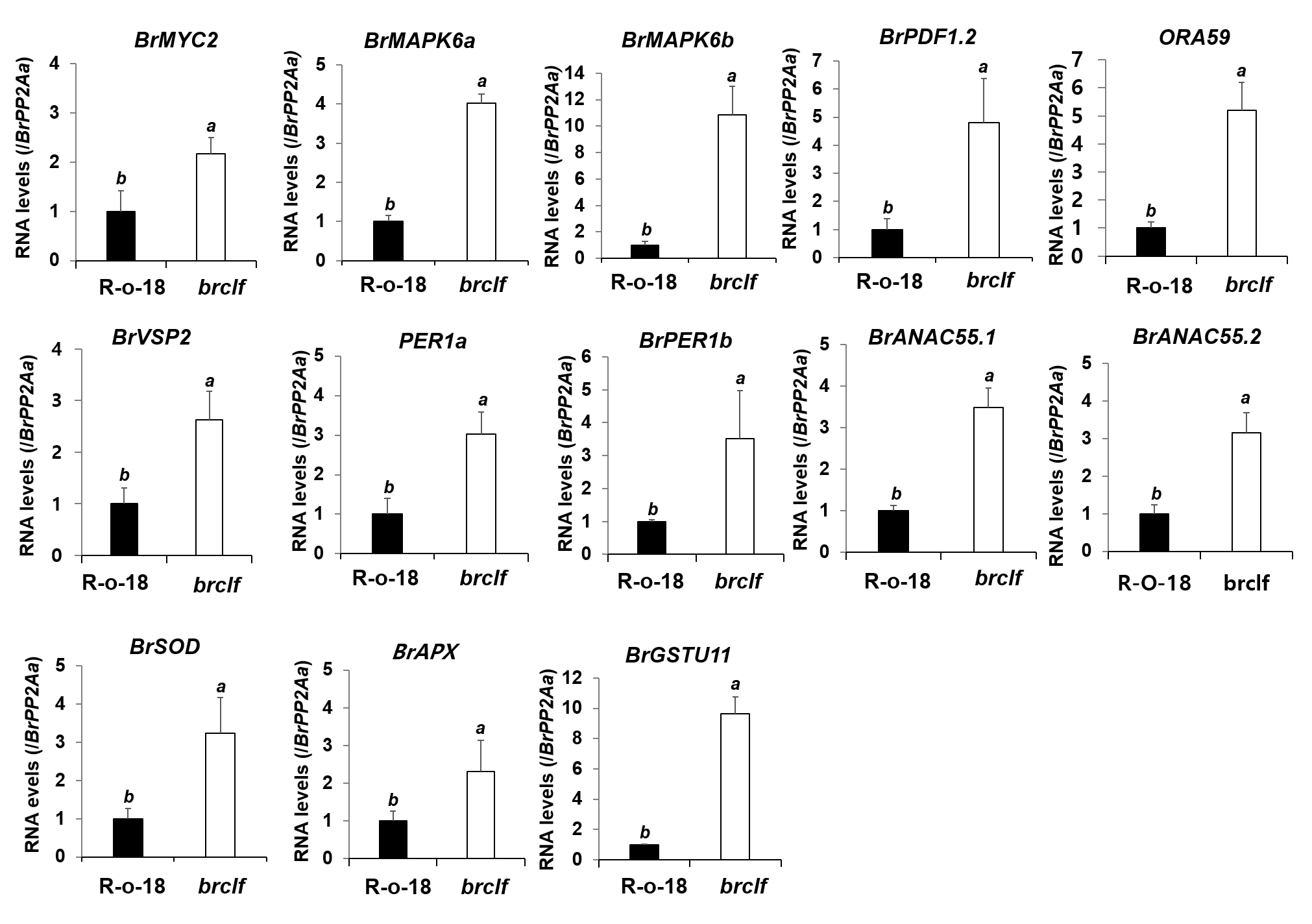

Supplement: Supplementary Figure 1 — Identification of loss of function mutant of BrCLF, brclf. (A) Multiple alignment of amino acid sequences of Arabidopsis CLF (AtCLF) clade and B. rapa homologous genes, (named BrCLF, BrSWN, BrMEA.a, and BrMEA.b). Catalytic SET domain for H3K27 (H3K27me3) trimethylation was indicated with a green box. (B) Phylogenic analysis of Arabidopsis CLF (AtCLF) clade and B. rapa homologous proteins (BrCLF, BrSWN, BrMEA.a, and BrMEA.b). AtCLF has a single B. rapa homolog, BrCLF (Bra032169, indicated with red letters). (C) Sanger sequencing validation of point mutation in 11th exon of BrCLF coding sequence in brclf mutant. Nucleotide at 1,843bp from the start codon of BrCLF was converted from C to T which generate a premature stop codon in brclf mutant. (D) Comparison of the full amino acid sequence of BrCLF of R-o-18 and brclf mutant. Glutamine (Gln) residue at 615th was converted to a stop codon (indicated with asterisk), generating the truncated form of BrCLF missing the H3K27me3 catalytic SET domain in the C-terminal region. [file DataSheet_1.zip › (rev)Supp Fig. S6.tif]

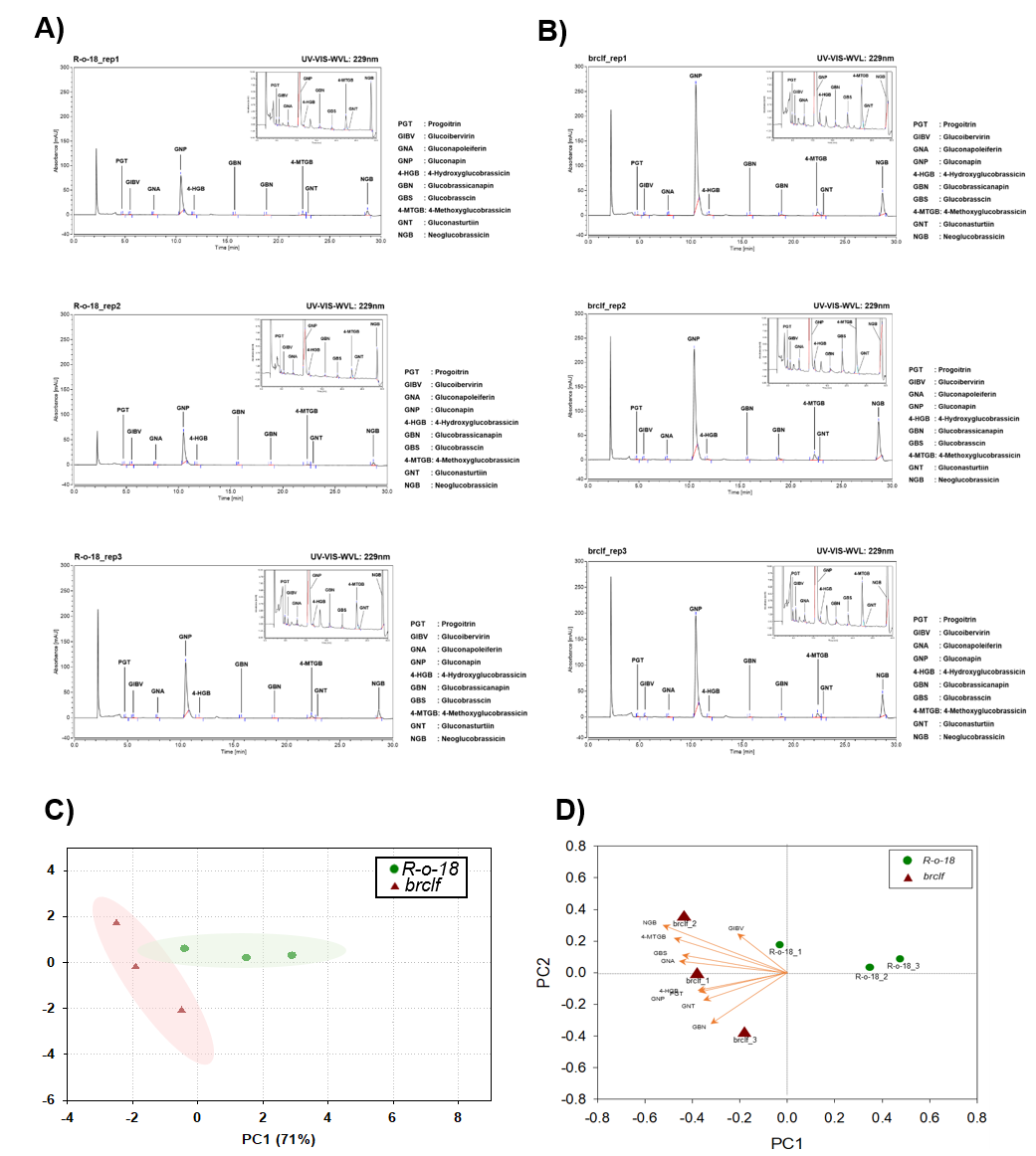

Supplement: Supplementary Figure 1 — Identification of loss of function mutant of BrCLF, brclf. (A) Multiple alignment of amino acid sequences of Arabidopsis CLF (AtCLF) clade and B. rapa homologous genes, (named BrCLF, BrSWN, BrMEA.a, and BrMEA.b). Catalytic SET domain for H3K27 (H3K27me3) trimethylation was indicated with a green box. (B) Phylogenic analysis of Arabidopsis CLF (AtCLF) clade and B. rapa homologous proteins (BrCLF, BrSWN, BrMEA.a, and BrMEA.b). AtCLF has a single B. rapa homolog, BrCLF (Bra032169, indicated with red letters). (C) Sanger sequencing validation of point mutation in 11th exon of BrCLF coding sequence in brclf mutant. Nucleotide at 1,843bp from the start codon of BrCLF was converted from C to T which generate a premature stop codon in brclf mutant. (D) Comparison of the full amino acid sequence of BrCLF of R-o-18 and brclf mutant. Glutamine (Gln) residue at 615th was converted to a stop codon (indicated with asterisk), generating the truncated form of BrCLF missing the H3K27me3 catalytic SET domain in the C-terminal region. [file DataSheet_1.zip › (rev)Supp Fig. S7.tif]

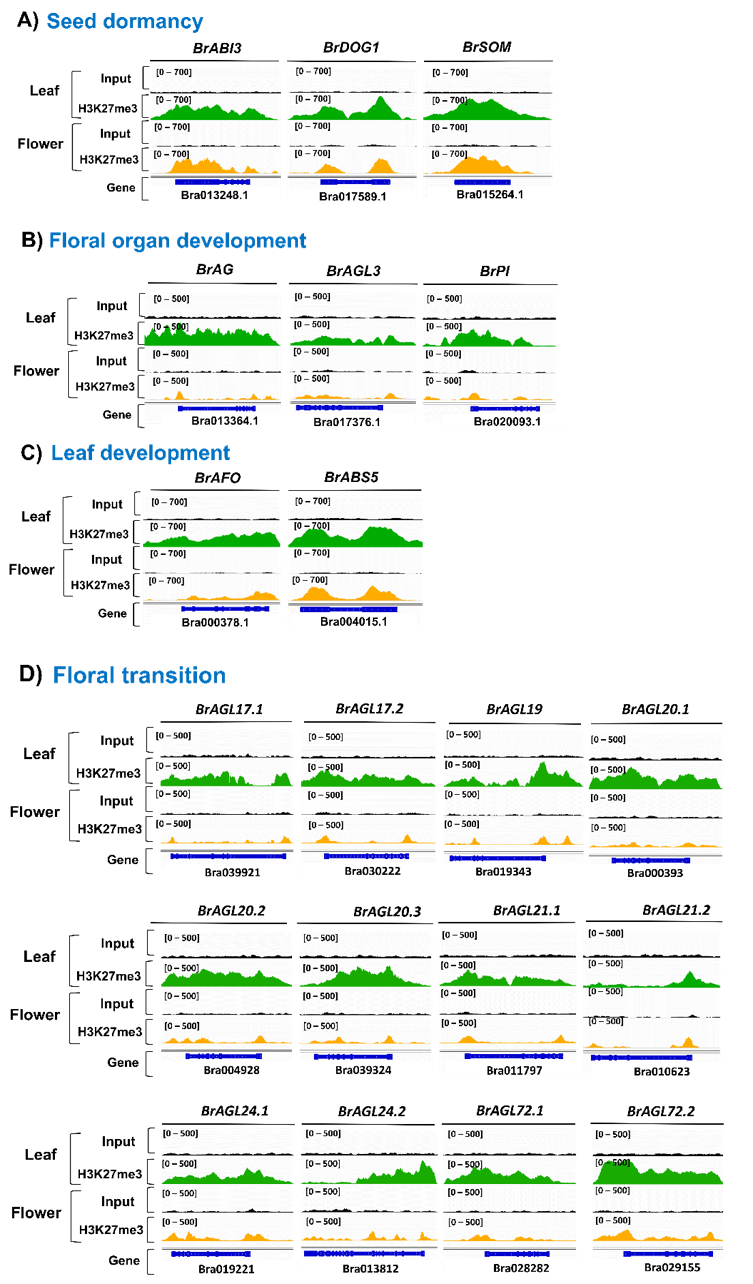

Supplement: Supplementary Figure 1 — Identification of loss of function mutant of BrCLF, brclf. (A) Multiple alignment of amino acid sequences of Arabidopsis CLF (AtCLF) clade and B. rapa homologous genes, (named BrCLF, BrSWN, BrMEA.a, and BrMEA.b). Catalytic SET domain for H3K27 (H3K27me3) trimethylation was indicated with a green box. (B) Phylogenic analysis of Arabidopsis CLF (AtCLF) clade and B. rapa homologous proteins (BrCLF, BrSWN, BrMEA.a, and BrMEA.b). AtCLF has a single B. rapa homolog, BrCLF (Bra032169, indicated with red letters). (C) Sanger sequencing validation of point mutation in 11th exon of BrCLF coding sequence in brclf mutant. Nucleotide at 1,843bp from the start codon of BrCLF was converted from C to T which generate a premature stop codon in brclf mutant. (D) Comparison of the full amino acid sequence of BrCLF of R-o-18 and brclf mutant. Glutamine (Gln) residue at 615th was converted to a stop codon (indicated with asterisk), generating the truncated form of BrCLF missing the H3K27me3 catalytic SET domain in the C-terminal region. [file DataSheet_1.zip › (rev)Supp Fig. S8.tif]

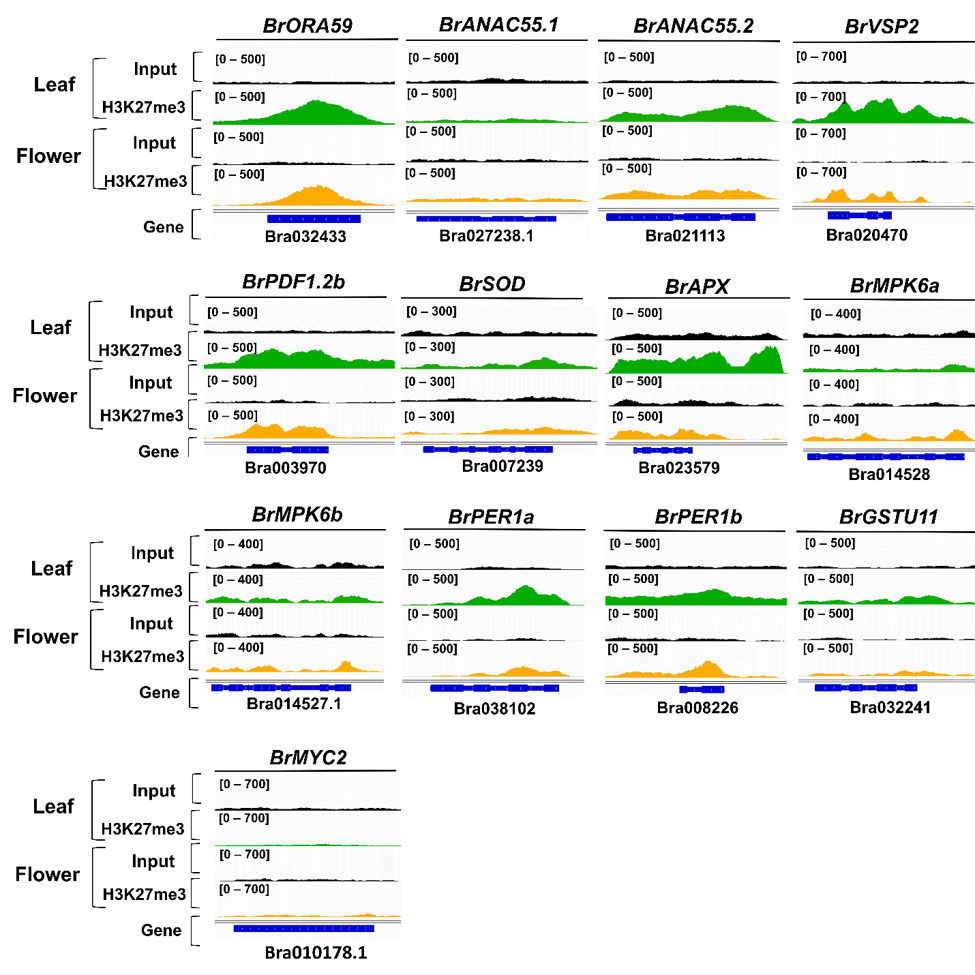

Supplement: Supplementary Figure 1 — Identification of loss of function mutant of BrCLF, brclf. (A) Multiple alignment of amino acid sequences of Arabidopsis CLF (AtCLF) clade and B. rapa homologous genes, (named BrCLF, BrSWN, BrMEA.a, and BrMEA.b). Catalytic SET domain for H3K27 (H3K27me3) trimethylation was indicated with a green box. (B) Phylogenic analysis of Arabidopsis CLF (AtCLF) clade and B. rapa homologous proteins (BrCLF, BrSWN, BrMEA.a, and BrMEA.b). AtCLF has a single B. rapa homolog, BrCLF (Bra032169, indicated with red letters). (C) Sanger sequencing validation of point mutation in 11th exon of BrCLF coding sequence in brclf mutant. Nucleotide at 1,843bp from the start codon of BrCLF was converted from C to T which generate a premature stop codon in brclf mutant. (D) Comparison of the full amino acid sequence of BrCLF of R-o-18 and brclf mutant. Glutamine (Gln) residue at 615th was converted to a stop codon (indicated with asterisk), generating the truncated form of BrCLF missing the H3K27me3 catalytic SET domain in the C-terminal region. [file DataSheet_1.zip › (rev)Supp Fig. S9.tif]

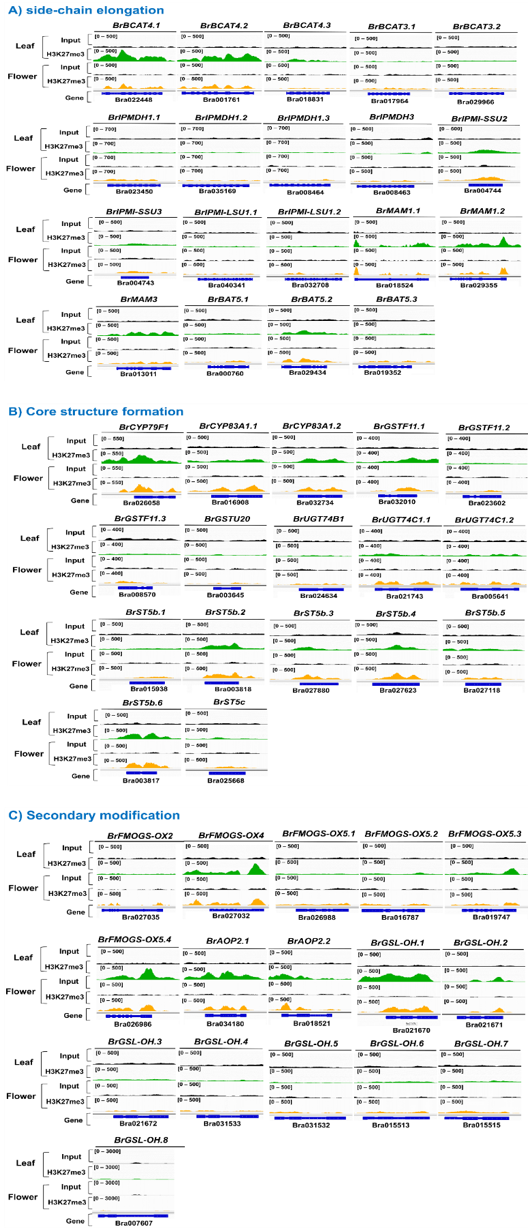

Supplement: Supplementary Figure 1 — Identification of loss of function mutant of BrCLF, brclf. (A) Multiple alignment of amino acid sequences of Arabidopsis CLF (AtCLF) clade and B. rapa homologous genes, (named BrCLF, BrSWN, BrMEA.a, and BrMEA.b). Catalytic SET domain for H3K27 (H3K27me3) trimethylation was indicated with a green box. (B) Phylogenic analysis of Arabidopsis CLF (AtCLF) clade and B. rapa homologous proteins (BrCLF, BrSWN, BrMEA.a, and BrMEA.b). AtCLF has a single B. rapa homolog, BrCLF (Bra032169, indicated with red letters). (C) Sanger sequencing validation of point mutation in 11th exon of BrCLF coding sequence in brclf mutant. Nucleotide at 1,843bp from the start codon of BrCLF was converted from C to T which generate a premature stop codon in brclf mutant. (D) Comparison of the full amino acid sequence of BrCLF of R-o-18 and brclf mutant. Glutamine (Gln) residue at 615th was converted to a stop codon (indicated with asterisk), generating the truncated form of BrCLF missing the H3K27me3 catalytic SET domain in the C-terminal region. [file DataSheet_1.zip › (rev)Supp Fig.11.tif]

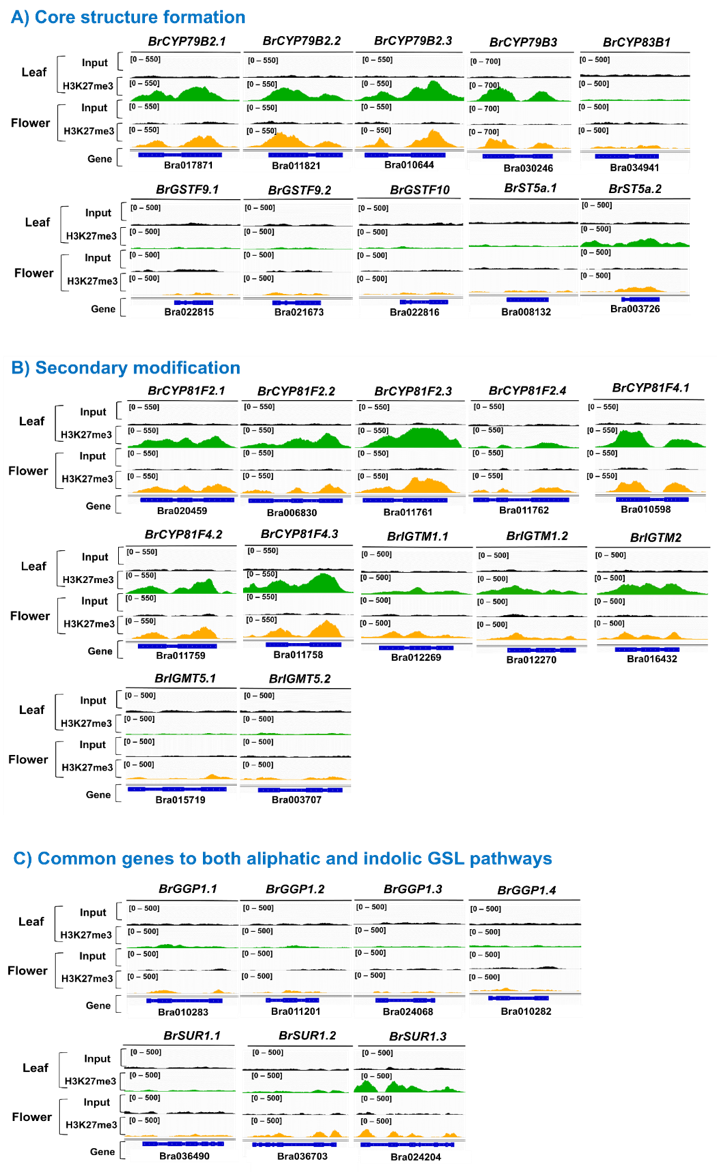

Supplement: Supplementary Figure 1 — Identification of loss of function mutant of BrCLF, brclf. (A) Multiple alignment of amino acid sequences of Arabidopsis CLF (AtCLF) clade and B. rapa homologous genes, (named BrCLF, BrSWN, BrMEA.a, and BrMEA.b). Catalytic SET domain for H3K27 (H3K27me3) trimethylation was indicated with a green box. (B) Phylogenic analysis of Arabidopsis CLF (AtCLF) clade and B. rapa homologous proteins (BrCLF, BrSWN, BrMEA.a, and BrMEA.b). AtCLF has a single B. rapa homolog, BrCLF (Bra032169, indicated with red letters). (C) Sanger sequencing validation of point mutation in 11th exon of BrCLF coding sequence in brclf mutant. Nucleotide at 1,843bp from the start codon of BrCLF was converted from C to T which generate a premature stop codon in brclf mutant. (D) Comparison of the full amino acid sequence of BrCLF of R-o-18 and brclf mutant. Glutamine (Gln) residue at 615th was converted to a stop codon (indicated with asterisk), generating the truncated form of BrCLF missing the H3K27me3 catalytic SET domain in the C-terminal region. [file DataSheet_1.zip › (rev)Supp Fig.12.tif]

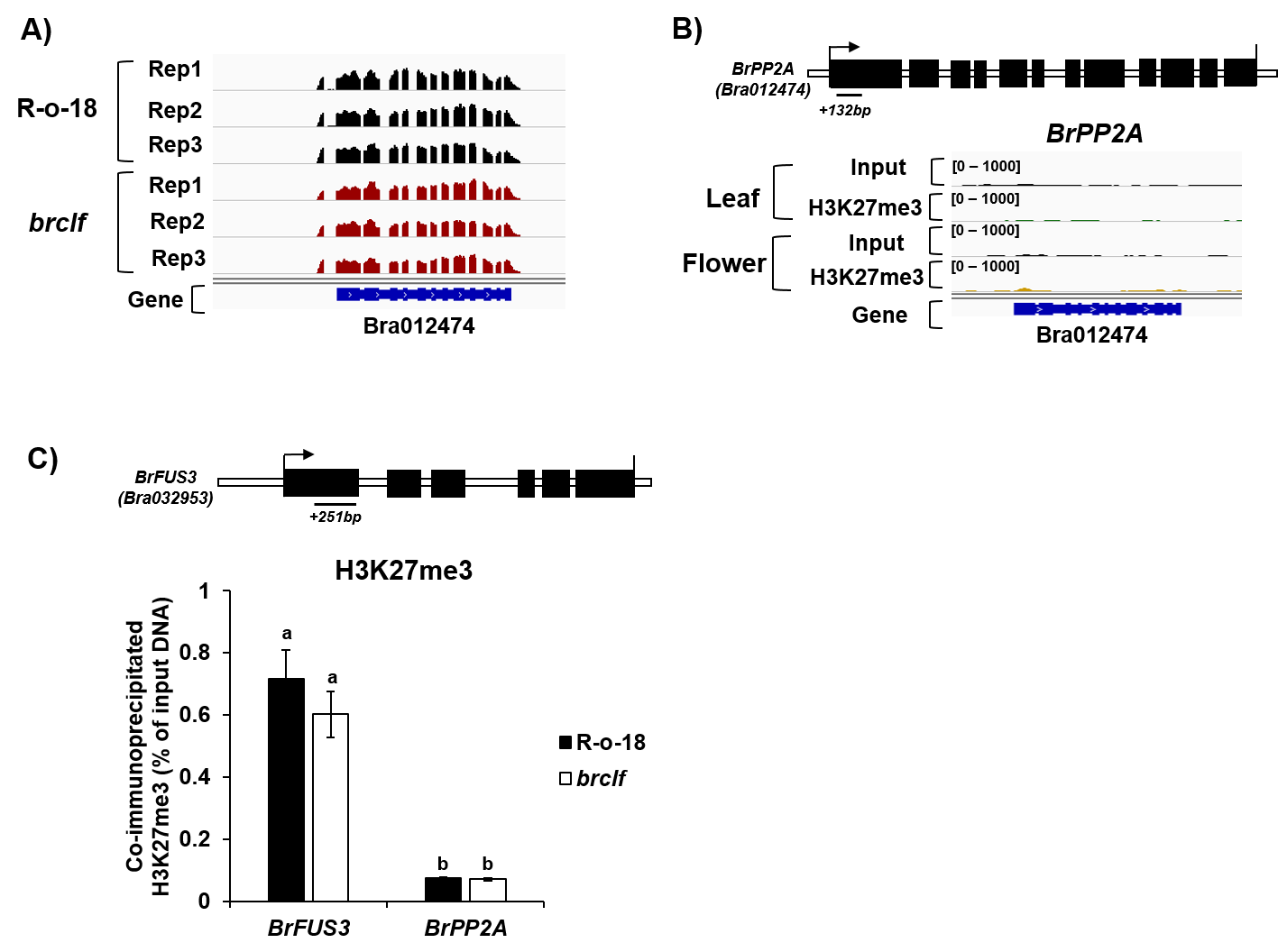

Supplement: Supplementary Figure 1 — Identification of loss of function mutant of BrCLF, brclf. (A) Multiple alignment of amino acid sequences of Arabidopsis CLF (AtCLF) clade and B. rapa homologous genes, (named BrCLF, BrSWN, BrMEA.a, and BrMEA.b). Catalytic SET domain for H3K27 (H3K27me3) trimethylation was indicated with a green box. (B) Phylogenic analysis of Arabidopsis CLF (AtCLF) clade and B. rapa homologous proteins (BrCLF, BrSWN, BrMEA.a, and BrMEA.b). AtCLF has a single B. rapa homolog, BrCLF (Bra032169, indicated with red letters). (C) Sanger sequencing validation of point mutation in 11th exon of BrCLF coding sequence in brclf mutant. Nucleotide at 1,843bp from the start codon of BrCLF was converted from C to T which generate a premature stop codon in brclf mutant. (D) Comparison of the full amino acid sequence of BrCLF of R-o-18 and brclf mutant. Glutamine (Gln) residue at 615th was converted to a stop codon (indicated with asterisk), generating the truncated form of BrCLF missing the H3K27me3 catalytic SET domain in the C-terminal region. [file DataSheet_1.zip › (rev)Supp Fig.S13.tif]
